# Development of translational read-through-inducing drugs as novel therapeutic options for patients with Fanconi anemia

**DOI:** 10.1038/s41420-025-02571-0

**Published:** 2025-06-21

**Authors:** Anca Manuela Hristodor, Enrico Cappelli, Elena Baldisseri, Roberto Valli, Giuseppe Montalbano, Giovanni Micheloni, Giovanni Porta, Annalisa Frattini, Silvia Ravera, Francesca Fioredda, Giuseppe Lippi, Carlo Dufour, Marco Cipolli, Valentino Bezzerri

**Affiliations:** 1 Cystic Fibrosis Center, Azienda Ospedaliera Universitaria Integrata, Verona, Italy; 2 Unit of Hematology, IRCCS G. Gaslini, Genoa, Italy; 3 Department of Medicine and Surgery, University of Insubria, Varese, Italy; 4 Genomic Medicine Center, University of Insubria, Varese, Italy; 5 Institute for Genetic and Biomedical Research (IRGB), Consiglio Nazionale delle Ricerche, Milan, Italy; 6 Department of Experimental Medicine, University of Genoa, Genoa, Italy; 7 Unit of Clinical Biochemistry, Department of Engineering for Innovation Medicine, University of Verona, Verona, Italy; 8 Department of Life Sciences, Health and Health Professions, Link Campus University, Rome, Italy

**Keywords:** Translational research, Drug development

## Abstract

Fanconi anemia (FA) is caused by mutations affecting FANC genes involved in DNA repair, with nearly 20% of FA patients harboring nonsense mutations. Ataluren (PTC124) is a translational read-through-inducing drug (TRID) already approved in Europe that has a well-established safety profile even in pediatric patients. Amlexanox, an anti-inflammatory drug, also promotes read-through of premature stop codons caused by nonsense mutations. We compared ataluren and amlexanox in rescuing FANCA, FANCC and FANCF protein synthesis in lymphoblastoid cell lines and fibroblasts obtained from FA patients with nonsense mutations. While ataluren restored all FANC protein levels, amlexanox was partially effective only on FANCA. Notably, the rescue of FANC proteins resulted in a significant downregulation of p53. Moreover, unlike amlexanox, ataluren remarkably improved cell viability and reduced chromosomal aberrations upon exposure to genotoxic compounds. Amlexanox primarily reduced the signal transducer and activator of transcription 2 (STAT2) phosphorylation. Furthermore, *FANCA*-mutated fibroblasts exhibited a higher frequency of micronuclei formation as well as lower lamin B1 expression compared to their gene-edited counterpart re-expressing wild-type *FANCA*. Interestingly, ataluren significantly limited the generation of micronuclei in nonsense-mutated primary *FANCC* fibroblasts, restoring lamin B1 expression. This study represents a milestone of drug development for FA as it paves the way for clinical development of TRIDs, indicating ataluren as a promising approach to address the genetic instability and reduce the risk of malignant transformation in FA cells. Moreover, these results highlight the importance of a reliable experimental pipeline to assess whether minimal protein rescue via translational read-through can yield meaningful phenotypic rescue.

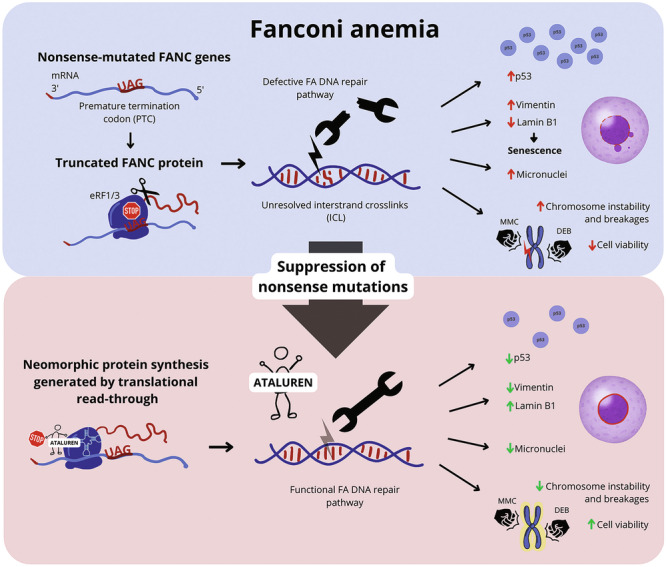

## Background

Fanconi anemia (FA) is a genetically diverse disorder caused by mutations in at least 22 identified Fanconi Anemia Complementation group (FANC) genes [1], which play critical roles in repairing DNA interstrand crosslinks (ICL) that threaten genomic integrity.

Dysfunctional FANC proteins lead to a progressive accumulation of DNA damage, particularly in replicating cells. Notably, loss of *FANCA* and *FANCC* expression has been associated with the formation of micronuclei in bone marrow aspirates from FA patients and in transgenic mice. Micronuclei formation, attributed to deficient DNA-damage response (DDR) in FA cells, has been proposed as causative of chromosome breakage [2]. Lamin B1, a key nuclear lamina component, plays a pivotal role in the formation of these structures, as its loss promotes nuclear envelope collapse and thus, micronuclei generation [3]. Micronuclei are recognized by the cyclic GMP-AMP Synthase (cGAS) - Stimulator of Interferon Genes (STING) pathway, triggering a cell-intrinsic surveillance process that controls neoplastic transformation [4]. In addition, persistent DNA repair failure promotes the activation of tumor suppressor protein p53, resulting in cell cycle arrest and increased apoptosis [5]. In the bone marrow (BM) compartment, this process culminates in bone marrow failure (BMF) and increased predisposition to hematological malignancy.

*FANCA* is the most commonly mutated gene, accounting for 65% of all FA cases, with other mutations occurring less frequently [6]. According to the ClinVar database [7], 16%, 21%, and 31% of pathogenic and likely-pathogenic variants of *FANCA*, *FANCC*, and *FANCF*, respectively, are nonsense mutations. These mutations result in premature termination codons (PTC), often producing truncated proteins or triggering the activation of nonsense-mediated decay (NMD) of mutated transcripts. Innovative therapeutic approaches aim at forcing the translational read-through of the PTC, by substituting the recruitment of the eukaryotic translation termination complex eRF1/eRF3 with the insertion of a near-cognate tRNA [8]. NMD inhibition, targeting the up-frameshift suppressor 1 homolog (UPF1) and the nonsense-mediated mRNA decay-associated PI3K-related kinase (SMG1) factors [9], is another approach to suppress nonsense mutations. Among translational read-through-inducing drugs (TRID), ataluren (PTC124) has shown promising results in restoring several nonsense-mutated genes, both in vitro and in vivo [10–14]. Additionally, it has been clinically tested in hundreds of patients with Duchenne muscular dystrophy (DMD) [15, 16] and cystic fibrosis [17, 18], demonstrating an excellent safety profile. Recently, amlexanox, an anti-inflammatory drug approved for treating aphthous ulcers, has been identified as a dual-activity molecule able to simultaneously act as a TRID and NMD inhibitor [19, 20].

The only treatment currently available for BMF in FA subjects is allogeneic hematopoietic stem cell transplantation (HSCT). However, HSCT does not prevent the occurrence of solid tumors and FA patients face high risks due to their genetic instability and extreme sensitivity to DNA crosslinking agents used in standard conditioning regimens [21]. This underscores an urgent unmet need for pharmaceutical therapies aimed at promoting genetic stability and reducing predisposition to malignant transformation in FA. Given the high incidence of nonsense mutations in inherited bone marrow failure syndromes (IBMFS), we tested the effect of ataluren and amlexanox in restoring nonsense-mutated *FANCA* and *FANCF* gene expression in patient-derived lymphoblastoid cell lines (LCL), as well as the subsequent effects on FANC-dependent DNA damage, chromosome rearrangements and *TP53* expression. The formation of micronuclei was also investigated in primary fibroblasts obtained from patients harboring nonsense mutations on *FANCC*.

## Results

### Effect of TRIDs on full-length FANCA and FANCF neomorphic protein synthesis in LCL

**Fig. 1:**
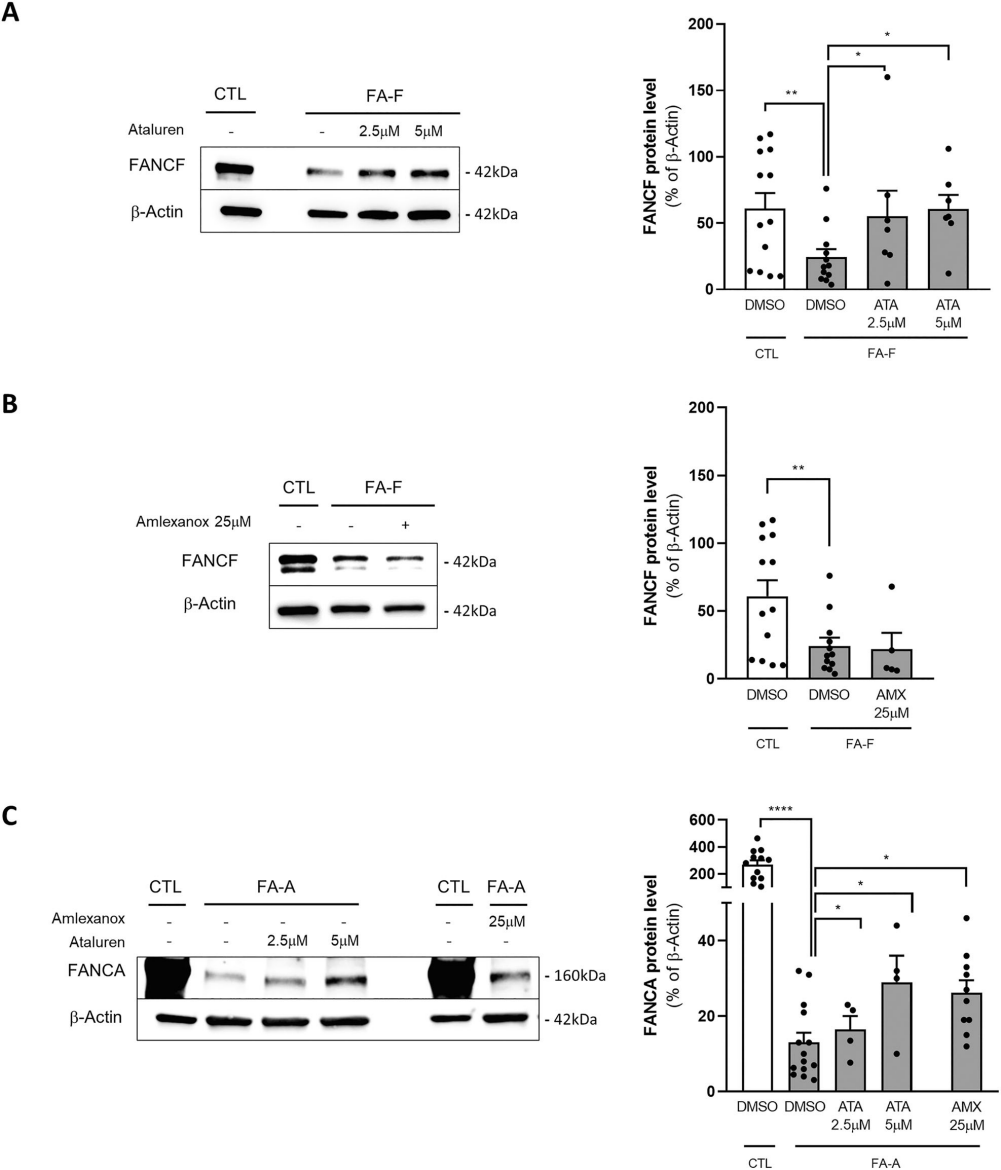
Ataluren significantly increased both FANCF and FANCA protein expression, while amlexanox rescued only FANCA synthesis. Representative Western blots (left panels) and corresponding protein quantification (right panels) after 24 h incubation with ataluren (2.5-5 μM) or amlexanox (25 μM) of FANCF (**A**, *n* = 7; **B**, *n* = 4) and FANCA (**C**, *n* = 5), in FA-F and FA-A mutated LCL, respectively. Data are mean ± SEM. The Shapiro-Wilk test was used to calculate normality, followed as appropriate by a nonparametric Wilcoxon test or paired *t* test (*p < 0.05; **p < 0.01; ****p < 0.0001).

We recently described the beneficial effect of ataluren on the restoration of Shwachman-Bodian-Diamond syndrome (SBDS) protein synthesis in various in vitro and ex vivo cell models of Shwachman-Diamond syndrome (SDS) [14, 22]. On this basis, we sought to evaluate the effect of ataluren in restoring FANCA and FANCF full-length protein synthesis in LCL obtained from patients with FA. Most of these patients show biallelic mutations in *FANCA*, whereas a very limited number display mutations in FANCF. We were thus able to obtain four *FANCA*-mutated (FA-A) and one *FANCF*-mutated (FA-F) LCL models, all carrying nonsense mutations (Supplementary Table 1). We incubated LCL with 2.5 and 5 *μ*M ataluren, or 25 *μ*M amlexanox, or DMSO (vehicle) for 24h [20, 22]. FANCA and FANCF protein levels were evaluated by Western blot analysis. When untreated, FA-F cells showed almost 40% of FANCF protein levels compared with healthy control cells (Fig. 1A). After ataluren treatment, FANCF protein levels were similar to healthy controls. On the contrary, we observed no effect on FANCF protein levels following amlexanox treatment in these cells (Fig. 1B). FANCA levels were greatly reduced in FA-A LCL, to just under 5% of healthy controls (Fig. 1C). In this case, both ataluren and amlexanox were able to significantly induce FANCA full-length protein synthesis to almost 11% and 9% of healthy controls, respectively (Fig. 1C). Since amlexanox has also been proposed as an NMD inhibitor, we checked its effect on *FANCA* mRNA expression in FA-A cells. We observed that NMD is activated in these cells, resulting in a 3-fold reduction of *FANCA* mRNA. However, neither amlexanox nor ataluren was able to rescue *FANCA* transcription (Supplementary Fig. 1A, B). Interestingly, NMD was not activated in FA-F cells, which indeed show similar levels of transcripts compared to healthy control cell lines (Supplementary Fig. 1C). This may explain why we observed more basal FANCF protein levels than FANCA in our cell models.

### Both ataluren and amlexanox reduce p53 levels in FA cells

**Fig. 2:**
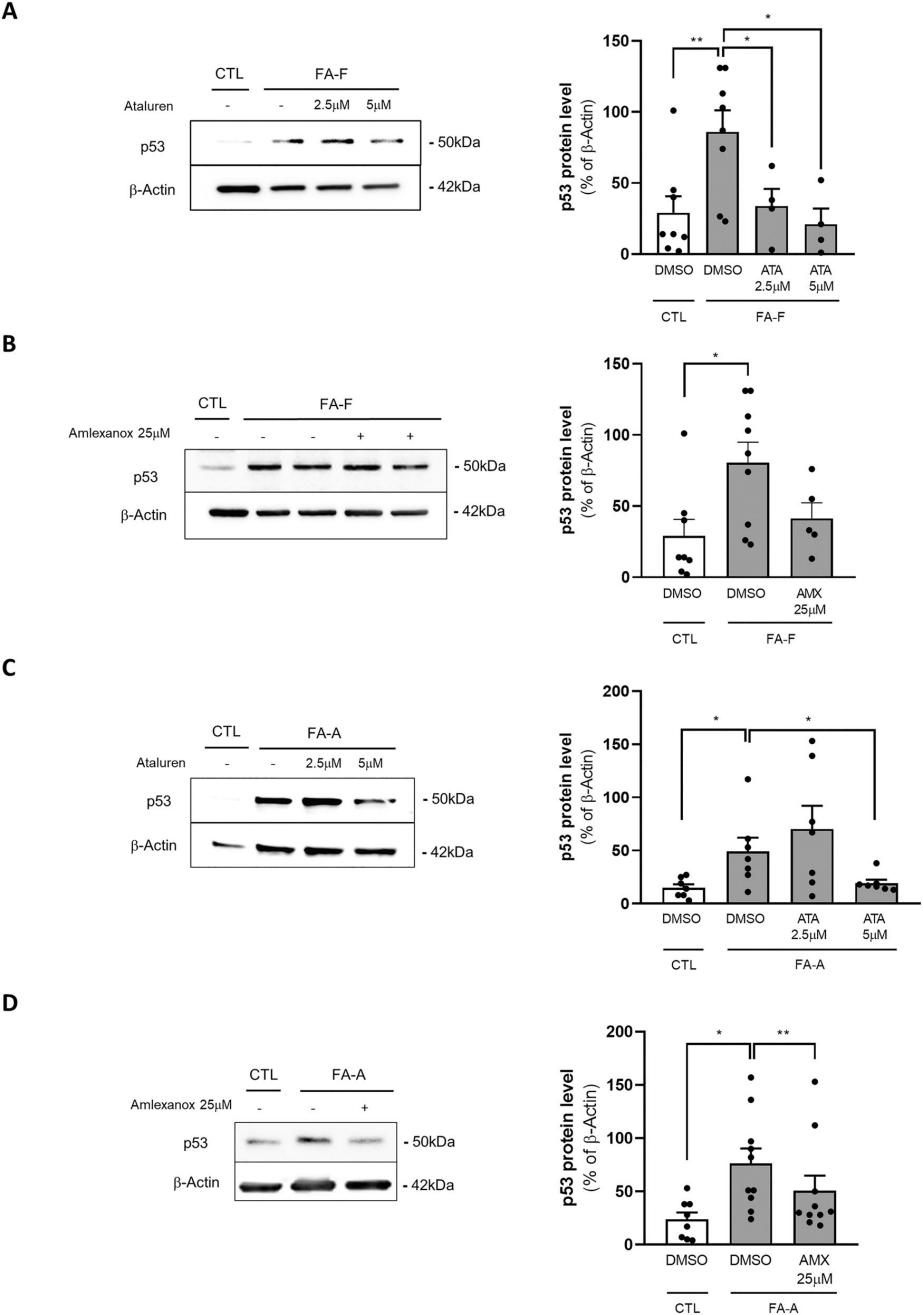
Ataluren significantly reduced p53 expression levels in FA-F and FA-A mutated LCLs, while amlexanox reduced p53 solely in FA-A LCL. Representative Western blots (left panels) and corresponding protein quantification (right panels) of p53 in FA-F mutated LCL after 24 h incubation with ataluren at 2.5μM and 5μM (**A**, *n* = 4); after 24 h incubation with amlexanox 25μM (**B**, *n* = 5); and in FA-A after ataluren (**C**, *n* = 7) and amlexanox (**D**, *n* = 10). Data are mean ± SEM. The Shapiro-Wilk test was used to calculate normality, followed as appropriate by a nonparametric Wilcoxon test or paired *t* test (*p < 0.05; **p < 0.01).

The defective DNA repair mechanism associated with FA drives the activation of p53, which, in turn, leads to the downregulation of 9 FANC genes [23], eventually increasing the risk for chromosomal aberrations. Importantly, the hematopoietic defects in FA can be rescued by knocking down *tp53* expression in the bone marrow of *Fanca* and *Fancd2* mutant mice [5, 24]. We therefore sought to determine whether ataluren and amlexanox were able to reduce p53 levels in our experimental models. We found that 5 µM ataluren significantly reduced p53 levels both in FA-A and in FA-F cells (Fig. 2A–C), showing levels similar to healthy controls, whereas amlexanox exhibited a weaker effect, reducing almost 40% of p53 protein accumulation only in FA-A cells (Fig. 2B–D). These results are consistent with the lack of efficacy of amlexanox on full-length FANCF protein synthesis restoration reported above.

### Ataluren, but not amlexanox, mitigates genotoxic damage induced by mitomycin C and 1,3 butadiene diepoxide in FA cells

**Fig. 3:**
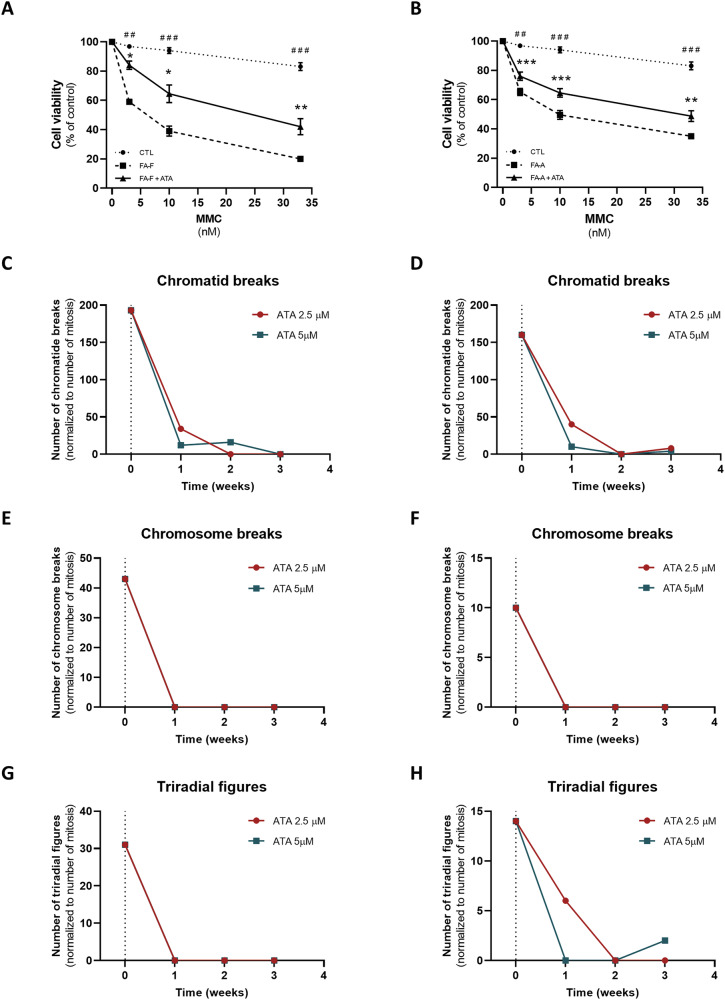
Ataluren significantly improved cell survival upon genotoxic treatment while limiting chromosomal aberrations. Mitomycin C test of FA-F (**A**, *n* = 4) and FA-A (**B**, *n* = 7) LCL, after 24 h incubation with ataluren 5μM. DEB test of FA-F (**C**, **E**, **G**) and FA-A (**D**, **F**, **H**) LCL, after 1, 2 and 3 weeks of incubation with ataluren 2.5μM (in red) and 5μM (in blue). Data for the MMC test are mean ± SEM. The Shapiro–Wilk test was used to calculate normality, followed by a parametric *t* test with *p* calculated accordingly (*p < 0.05; **p < 0.01; ***p < 0.001). *, comparison between basal FA condition and treated FA condition; #, comparison between healthy donors and FA.

**Fig. 4:**
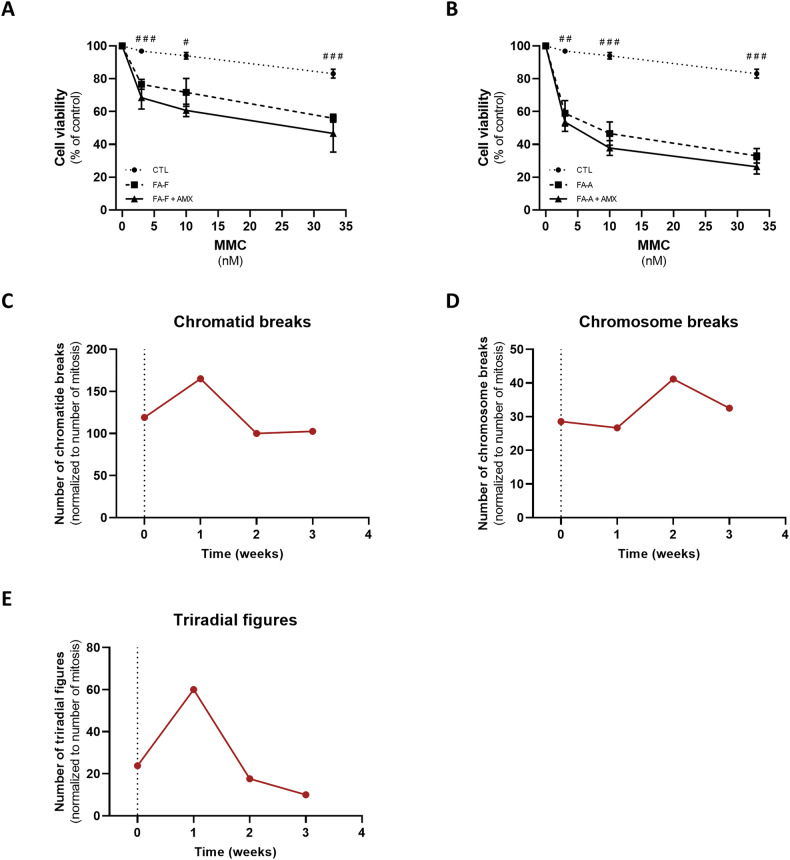
Amlexanox was not able to improve cell survival upon genotoxic treatment. Mitomycin C test of FA-F (**A**) and FA-A (**B**) LCL, after 24 h incubation with amlexanox 25μM (*n* = 3). DEB test after 1, 2 and 3 weeks of incubation with amlexanox 25μM (in red) of FA-A LCL (**C**–**E**). MMC test data are mean ± SEM. The Shapiro–Wilk test was used to calculate normality, followed by a parametric t test with *p* calculated accordingly (^#^*p* < 0.05; ^##^*p* < 0.01; ^###^*p* < 0.001). #, comparison between healthy donors and FA.

The current standard diagnostic practice for FA is testing for DNA hypersensitivity to clastogenic and crosslinking agents, including DEB [25] and MMC [26]. FA-A and FA-F cells were pre-incubated with ataluren, then exposed to MMC or DEB, following the current diagnostic protocols. FA-F and FA-A cells showed a significant decrease of up to 76% and 58% in cell viability upon MMC treatment, respectively, displaying a dose-dependent pattern (Fig. 3A, B). Interestingly, ataluren induced up to a 2.1-fold increase in cell survival in *FANCF* mutants (Fig. 3A), whereas it promoted a 1.5-fold increase in cell viability in *FANCA* mutated LCL (Fig. 3B). DEB promoted chromosomal aberrations, including chromatid breaks, chromosome breakage, and triradial figures, both in FA-F and in FA-A cells. Remarkably, ataluren greatly ameliorated these forms of genetic damage, in a dose-dependent manner. Both concentrations of ataluren (2.5 and 5 *μ*M) showed similar results, almost completely depleting chromosome breakages and aberrations after three weeks of culture (Fig. 3C–H). Conversely, amlexanox showed no effect on either MMC- or DEB-induced genotoxicity in FA-A cells (Fig. 4), despite a slight increase in FANCA protein synthesis and a significant reduction of initial p53 levels.

### The effects of amlexanox on FA cells are not due to its anti-inflammatory properties, but rather linked to its TBK1/IKK*ε* inhibitory effect through the inhibition of pSTAT2

**Fig. 5:**
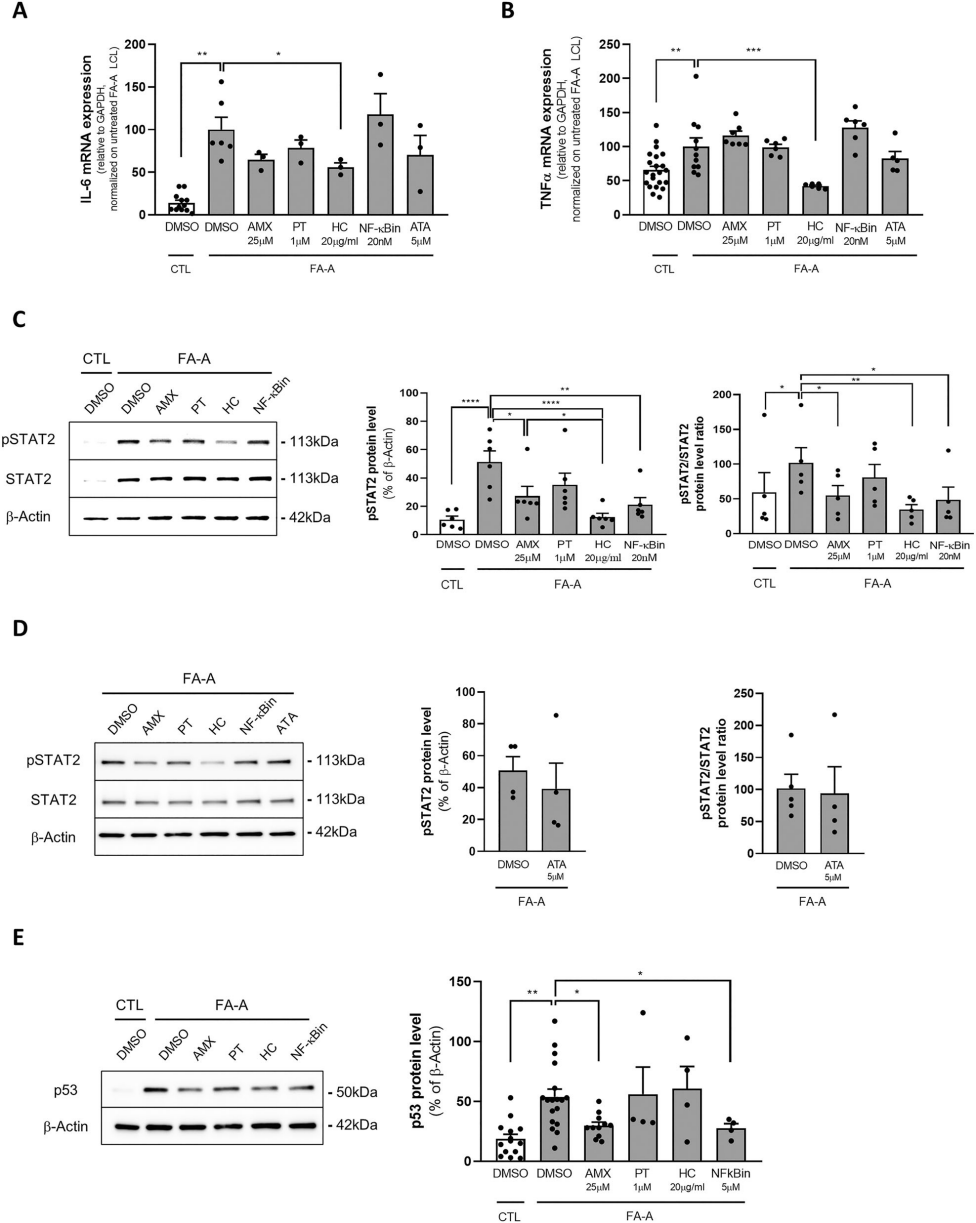
Amlexanox did not reduce the expression of pro-inflammatory markers but reduced the phosphorylation of STAT2. Pro-inflammatory cytokines IL-6 (**A**, *n* = 3) and TNFα (**B**, *n* = 6), and mRNA expression levels in FA-A mutated LCL, after 24 h incubation with amlexanox 25μM (AMX), parthenolide 1μM (PT), hydrocortisone 20μg/ml (HC), NF-**κ**B activation inhibitor 20 nM (NF-**κ**Bin), ataluren 5μM. pSTAT2 and STAT2 protein expression levels after AMX, PT, HC and NF-**κ**Bin (**C**, *n* = 5), and ATA (**D**, *n* = 4); expression of p53 (**E**, *n* = 4). Data are mean ± SEM. Welch’s ANOVA test (**A**, **B**), or mixed effect analysis (**C**, **E**) or one-way ANOVA (**D**) were used (*p < 0.05; **p < 0.01; ***p < 0.001; ****p < 0.0001).

Amlexanox was already described as an anti-inflammatory agent [27], with potential capabilities as TRID and NMD inhibitor [20, 28, 29]. Pro-inflammatory mediators, including tumor necrosis factor (TNF)-α [30] and IL-6 [31], are overexpressed in FA. We thus investigated if amlexanox promoted the reduction of p53 levels through its anti-inflammatory capability rather than the partial translational read-through of FANC transcripts. In order to address this issue, we performed qPCR to assess the mRNA expression of IL-6 and TNFα [30, 31]. We compared amlexanox with the following molecules: i) hydrocortisone, a broad anti-inflammatory drug; ii) parthenolide, a broad-range inhibitor of IkappaB kinase (IKK); iii) a selective nuclear factor kappa-light-chain-enhancer of activated B cell (NF-κB) inhibitor; iv) ataluren. FA-A LCL were treated for 24 hours with these compounds before quantifying cytokine transcripts. Amlexanox did not reduce *IL6* or *TNF*α expression, indicating that it does not exert a clear anti-inflammatory effect (Fig. 5A, B). Similarly, NF-κB inhibition mediated by both parthenolide (through IKK inactivation) and a selective NF-κB activation inhibitor did not reduce the expression of proinflammatory cytokines, suggesting that NF-κB is not a major driver in FA-related inflammation. As expected, a broad anti-inflammatory drug such as hydrocortisone significantly inhibited proinflammatory cytokine expression. Amlexanox is known to be a potent inhibitor of TANK-binding kinase 1 and nuclear factor kappa-B kinase subunit epsilon (TBK1/IKKε) complex [32–34], which can activate NF-κB and Interferon Regulatory Factor 3 (IRF3)/Interferon type I (IFN-I) signaling [35]. Since increasing evidence suggests the constitutive activation of the cGAS/STING/TBK1/IRF3 innate immune pathway in DNA-repair deficient cells and chromosome instability syndromes [35], including FA [36], we hypothesized that amlexanox may reduce p53 levels by mitigating the overactivation of TBK1/IKKε complex. The signal transducer and activator of transcription 2 (STAT2) is acknowledged as a major transcription factor-activated downstream IFN-I signaling pathway [37]. On this basis, we evaluated the phosphorylation levels of STAT2 (pSTAT2) in FA-A LCL. STAT2 was highly upregulated in FA-A LCL compared to healthy controls. In this model, amlexanox significantly reduced pSTAT2 levels (by almost 50%) after 24 h of incubation (Fig. 5C). Interestingly, treatment with NF-κB inhibitors resulted in similar pSTAT2 reduction, suggesting that the overall effect of amlexanox is mainly due to its inhibitory effects on the TBK1/IKKε pathway. An even greater inhibitory effect on pSTAT2 was observed upon incubation of FA cells with hydrocortisone, which has already been established as a broad JAK/STAT inhibitor [38], besides its possible direct inhibitory effect on the IRF3 pathway [39]. On the contrary, no significant effect on pSTAT2 was reported after ataluren incubation (Fig. 5D), strengthening the hypothesis that amlexanox and ataluren exert their effects through different mechanisms. Interestingly, NF-κB inhibitor significantly reduced p53 levels in FA cells, the degree of inhibition being similar to that observed with amlexanox treatment (Fig. 5E). These findings suggest that the effects of amlexanox are driven by NF-κB inhibition.

### Loss of lamin-B1 in the nuclear envelope leads to formation of micronuclei in FANCA- and FANCC-deficient cells, which can be rescued by ataluren treatment

**Fig. 6:**
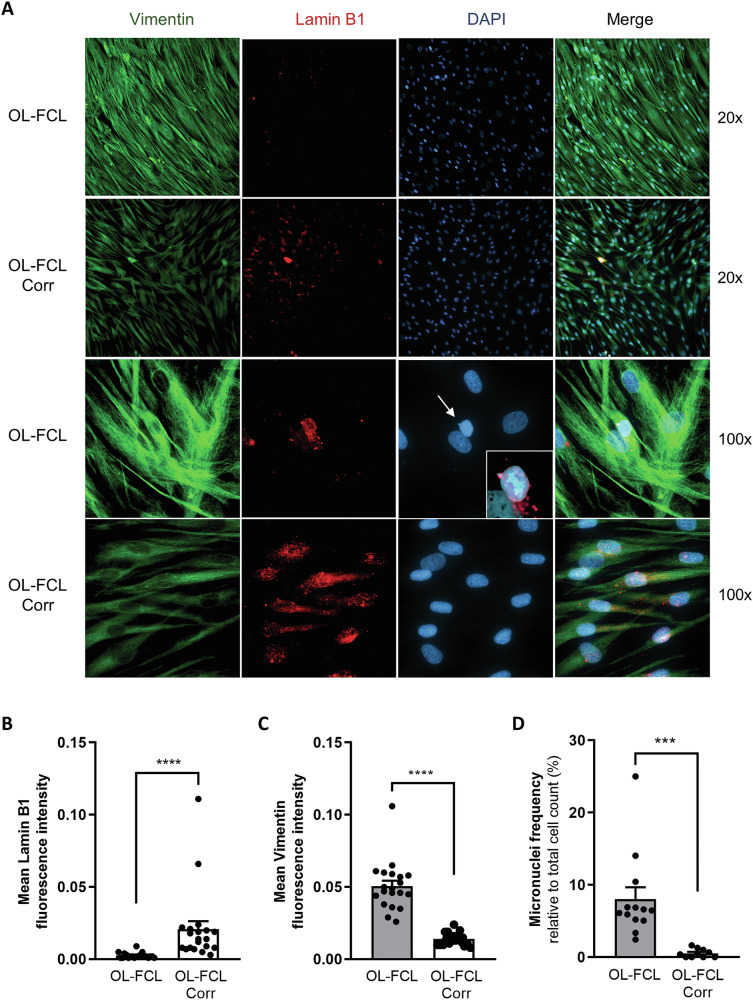
FA-A fibroblasts are characterized by reduced lamin B1, increased vimentin and formation of numerous micronuclei. Immunofluorescence staining of isogenic FANCA-mutant (OL-FCL) and FANCA*-*corrected (OL-FCL-Corr) fibroblasts for vimentin (green), lamin B1 (red) and DAPI (blue) (**A**). Quantification of fluorescence intensity of lamin B1 (**B**, *n* = 20) and vimentin (**C**, *n* = 20). Frequency of micronuclei in OL-FCL and OL-FCL-Corr fibroblasts was evaluated, counting almost 50 cells per field (**D**, *n* = 10 fields). Data are mean ± SEM. The Shapiro-Wilk test was used to calculate normality, followed as appropriate by a nonparametric Mann–Whitney test (**B**, **C**) or parametric *t* test (**D**) (***p < 0.001; ****p < 0.0001).

**Fig. 7:**
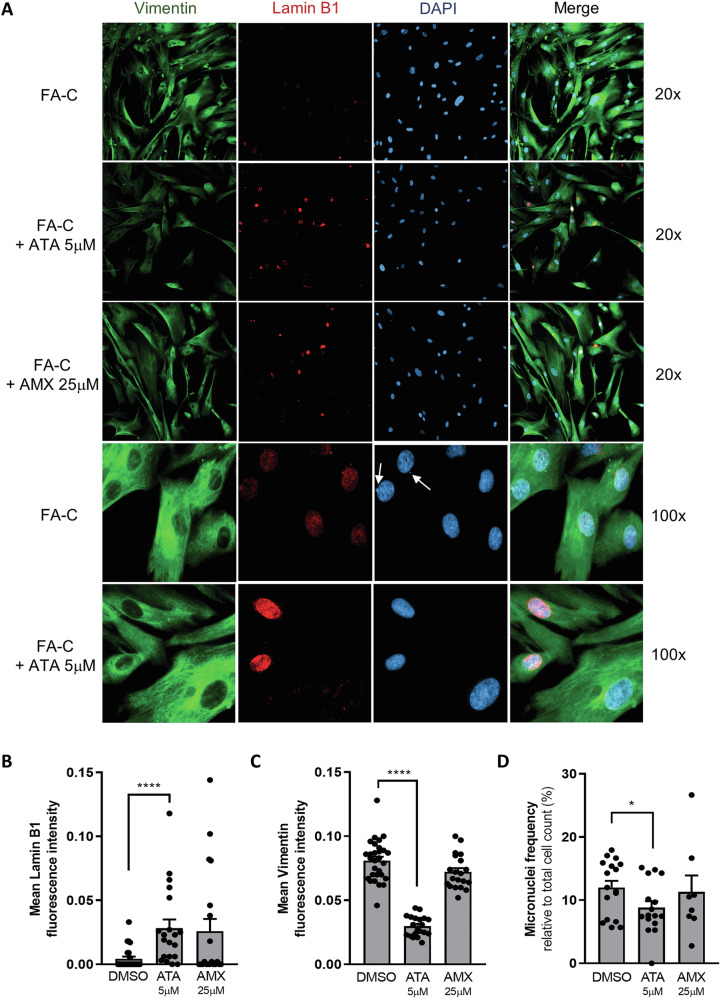
Ataluren increased lamin B1 levels, while reducing vimentin and micronuclei frequency in primary FA-C fibroblasts. Immunofluorescence staining of primary FA-C nonsense*-*mutated fibroblasts, before and after 24 h of incubation with ataluren 5μM or amlexanox 25μM, for vimentin (green), lamin B1 (red) and DAPI (blue) (**A**). Quantification of fluorescence intensity of lamin B1 (**B**, *n* = 20) and vimentin (**C**, *n* = 20). Frequency of micronuclei in FA-C fibroblasts before and after treatment with ataluren or amlexanox was evaluated by counting at least 50 cells per field (**D**, *n* = 8 fields). Data are mean ± SEM. The Shapiro-Wilk test was used to calculate normality, followed as appropriate by a nonparametric Mann–Whitney test (**B**) or parametric *t* test (**C**, **D**) (*p < 0.05; ****p < 0.0001).

Loss of FANCA has been associated with increased vimentin expression and loss of lamin A/C, which in turn promotes nuclear morphological defects [40]. An interplay between lamin A/C and lamin B1 exists, since they cooperate in maintaining the spatial organization of the genome within the nucleus, subsequently affecting the overall gene expression. Moreover, lamin A/C and lamin B1, together with other cytoskeletal components including vimentin, are all hallmarks of cellular senescence, a process already widely described in FA [41]. According to these findings, we observed increased levels of vimentin together with loss of lamin B1 levels in OL-FCL fibroblasts harboring biallelic mutations in *FANCA*, possibly due to senescent programs, such as p53 pathway activation (Fig. 6A–C). Engineered isogenic OL-FCL-Corr cells expressing wild-type FANCA showed increased levels of lamin B1 and reduced vimentin expression (Fig. 6A–C). Loss of lamin B1 in nuclear lamina has been associated with nuclear envelope collapse and formation of aberrant micronuclei [3]. Consistent with this association, we observed micronuclei in OL-FCL cells. Upon *FANCA* re-expression in corrected cells, micronuclei were almost completely lost (Fig. 6D and Supplementary Fig. 2). To validate these findings in a more appropriate cell model, we employed primary fibroblasts obtained from a patient harboring nonsense mutation in *FANCC* (FA-C). Consistent with data obtained from OL-FCL cells, most FA-C fibroblasts showed faint levels of lamin B1 associated with micronuclei (Fig. 7A–D), thus providing evidence that deficiency of most FANC proteins plays a pivotal role in nuclear architecture destabilization. Ataluren treatment showed a significant increase in FANCC full-length protein synthesis (Supplementary Fig. 3), leading to a significant 6.5-fold increase in lamin B1 synthesis and a 2.7-fold decrease in vimentin levels, followed by a significant reduction (−27%) of micronucleus formation in FANCC deficient cells even after 24 h of incubation (Fig. 7 and Supplementary Fig. 4). Again, amlexanox treatment showed no significant effects on micronuclei formation in these cells.

## Discussion

Since almost 20% of patients with FA carry nonsense mutations, we tested the effect of already known TRIDs, including ataluren and amlexanox, on the restoration of nonsense-mutated FANCA and FANCF protein synthesis. We observed a strong restoration of FANCF protein upon ataluren treatment, whereas a weaker effect of this drug was reported in *FANCA*-mutant cells. This may depend on the type of nonsense mutation tested, rather than greater selectivity of ataluren for FANCF. On the other hand, amlexanox improved only FANCA synthesis, inducing almost 9% of neomorphic protein compared with healthy donors. A partial explanation for the lower efficacy of amlexanox compared to ataluren might reside in the different molecular mechanism of the two drugs. Nevertheless, it should be noted that experience from Cystic Fibrosis highly effective modulator therapies revealed that a restoration of 10% of functional protein should be sufficient to significantly rescue the phenotype in inherited recessive diseases [42]. Consistently, similar findings have been observed in SDS cells upon treatment with ataluren [14]. Both ataluren and amlexanox reduced the exaggerated levels of p53 in FA cells. Of note, ataluren normalized p53 levels both in FA-A and FA-F cells, whereas amlexanox reduced p53 levels by almost 40% in FA-A cells. Since downregulation of p53 has been proposed as a therapeutic strategy for FA, we sought to evaluate whether a reduction of p53 levels promoted by TRIDs was paralleled by decreased DNA damage. Ataluren showed a 2.1-fold increase in cell viability upon genotoxic challenge (MMC) in *FANCF*-mutant cells, and a 1.4-fold increase in *FANCA* mutants. These results are consistent with the differential effect of ataluren on the restoration of FANCA and FANCF neomorphic protein synthesis. On the contrary, amlexanox did not promote any improvement in cell viability upon MMC treatment, despite its partial inhibitory effect on p53. Similar outcomes emerged from the DEB breakage test. Thus, this study suggests that a partial downregulation of p53 per se is not sufficient to produce phenotypic rescue in human cells.

**Fig. 8:**
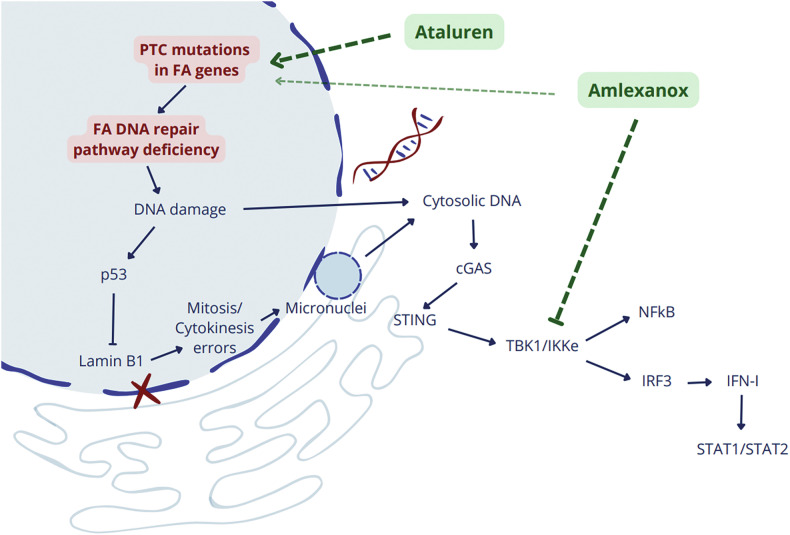
Proposed mechanism of action of ataluren and amlexanox. While both ataluren and amlexanox should induce translational read-through (and thus reduce the DNA damage repair deficiency, which would also result in reduced p53 levels), amlexanox is not able to improve cell survival upon genotoxic stress. There is admittedly a partial effect on FANCA protein expression and p53 reduction, indicating a broader spectrum of action. In Fanconi Anemia, amlexanox may have a stronger effect downstream of the DNA damage by inhibiting TBK1/IKKε, as already described in the literature, thus reducing the throughput of the IRF3/IFN-I and STAT2 pathway as well as NF-**κ**B activation. Of note, according to ENCODE data, *TP53* is a target gene of IRF3 transcription factor: this could be the main cause of the observed reduction in p53, which in this case does not translate into an overall improvement in cell fitness.

Amlexanox is also an already established anti-inflammatory drug. However, our results indicated that amlexanox did not have any anti-inflammatory effect in FA cells. Another general mechanism of action of amlexanox was attributed to its inhibitory effect on the TBK1/IKKε pathway, downstream cytosolic DNA sensing and cGAS/STING signaling [35]. Interestingly, here we reported the loss of lamin B1 in FANC-defective cells associated with the formation of micronuclei. These microstructures have been reported in a plethora of cancers and may further explain the high incidence of both solid and hematological malignancies in FA. Micronuclei may provide an explanation for cytosolic DNA sensing through pattern recognition receptors in FA. Against this background, our interpretation was that amlexanox reduced p53 levels through the inhibition of the cGAS/STING pathway. Accordingly, amlexanox significantly downregulated levels of pSTAT2, a transcription factor activated downstream cGAS/STING (Fig. 8). On the other hand, ataluren did not exhibit a significant effect on STAT2 phosphorylation. These findings strengthen our hypothesis that amlexanox and ataluren inhibited p53 expression through at least two different mechanisms. While ataluren reduces p53 accumulation because of an effective translational read-through, thus promoting the improvement of the DNA repair mechanism, amlexanox mainly acts downstream, inhibiting cGAS/STING signaling and STAT2.

Moreover, FA has already been established as a disease promoting cellular senescence [41]. Upregulation of the p53 pathway plays a key role in this process. We observed that FA cells have increased levels of the cytoskeletal protein vimentin and reduced levels of lamin B1, two hallmarks of senescence.

Importantly, besides improving lamin B1 synthesis, ataluren promoted a reduction of the exaggerated vimentin synthesis in FA cells, suggesting a wide spectrum of beneficial properties, including senomorphic capabilities. Most importantly, ataluren treatment significantly reduced micronuclei formation in primary *FANCC*-mutant fibroblasts carrying nonsense mutations.

In conclusion, this study represents a milestone of drug development for FA, indicating ataluren as a promising approach by which to address genetic instability and reduce the risk of malignant transformation in FA cells. These findings emphasize the need for a reliable experimental pipeline to assess whether the minimal amount of neomorphic protein synthesized upon translational read-through is actually followed by phenotypic rescue. Further studies examining the effect of ataluren and other effective TRIDs on hematopoiesis in bone marrow progenitor cells should confirm the possible usefulness of this therapeutic strategy in patients with FA. This would then pave the way for the clinical development of TRIDs in patients with FA.

## Materials and methods

### Cell cultures

*FANCA* (FA-A) mutated LCL, *FANCA* (OL-FCL) and *FANCC* (FA-C) mutated fibroblasts were provided by the Telethon Network Genetic Biobanks, Gaslini Hospital (Genoa, Italy). *FANCF* mutated (FA-F) LCL (GM16757) were obtained from the NIGMS Cell Repository at the Coriell Institute for Medical Research (Camden, NJ). LCL were cultured as cell suspensions in RPMI 1640 medium with 1% glutamine and 10% Fetal Bovine Serum (FBS, ThermoFisher, Waltham, MA), at 37 °C and 5% CO_2_. Fibroblasts were cultured in DMEM High Glucose (ThermoFisher) supplemented with 10% FBS and 1% glutamine. Cells were treated for 24 h with 2.5-5 µM ataluren, 25 µM amlexanox (Selleck Chemicals, Houston, TX), 20 µg/ml hydrocortisone (Selleck Chemicals), 1 μM parthenolide (Selleck Chemicals), 20 nM NF-κB activation inhibitor (Sigma-Aldrich, St. Louis, MA), or dimethyl sulfoxide (DMSO, Sigma-Aldrich) as a negative control, before protein or RNA extraction. OL-FCL fibroblasts have small and large deletions in *FANCA*. Isogenic OL-FCL corrected fibroblasts were generated by S11FAIN retrovirus [43]. Genetics of cell models employed in this study is reported in Supplementary Table 1.

### Western blot

Proteins were extracted in ice-cold RIPA buffer with protease inhibitors. 25 µg of total protein extracts in Laemmli buffer (cod.1610747, Bio-Rad, California, USA) were loaded onto 4–15% tris-glycine PAGE gels (cod. 4561084, Bio-Rad) and blotted onto nitrocellulose membranes (cod. 1704158, Bio-Rad) with a semidry method (Trans-Blot Turbo Transfer System, cod.1704150, Bio-Rad). Membranes were blocked for 1 hour in 5% skimmed milk in Tris-Buffered Saline (TBS) buffer with 0.1% Tween (TBS-T), and incubated overnight at +4 °C with primary antibodies: rabbit anti-FANCA (1:2000, ab264257, Abcam, Cambridge, UK), rabbit anti-FANCF (1:1000, ab47624, Abcam), rabbit anti-FANCC (1:1000, ab97575, Abcam), rabbit anti-p53 (1:1000, cod.2527, Cell Signaling Technology, Massachusetts, USA), rabbit anti-pSTAT2 (0.05μg/ml, MAB2890, R & D Systems, Minnesota, USA), anti-STAT2 (0.2 µg/mL, MAB1666-SP, R&D Systems), and mouse anti-β-Actin (1:10000, A5441, Sigma-Aldrich, Missouri, USA). Membranes were then washed with TBS-T and incubated for 1 h at room temperature with secondary HRP-conjugated antibodies: goat anti-rabbit (1:10000, cod.7074, Cell Signaling Technology) and goat anti-mouse (1:10000, cod.115-035-062, Jackson ImmunoResearch, Philadelphia, USA). Chemiluminescent detection (cod.1705062, Bio-Rad) was conducted by the ChemiDoc Imaging System (cod.12003153, Bio-Rad). All original western blot images are included in Original Data.

### qPCR

Total RNA was extracted and purified using the High Pure RNA Isolation Kit (Roche, Mannheim, Germany), then reverse transcribed into cDNA, using the High-Capacity cDNA Reverse Transcription Kit (ThermoFisher) in accordance with the manufacturers’ protocols. A total of 25 ng of cDNA was amplified using PowerUp Master Mix SYBR Green (ThermoFisher) and QuantiTect Primer Assays (Qiagen, Hilden, Germany) for *GAPDH* (Hs_GAPDH_1_SG, NM_001256799), *FANCA* (Hs_FANCA_1_SG, NM_000135, NM_001286167), *FANCF* (Hs_FANCF_1_SG, NM_022725), *IL6* (Hs_IL6_1_SG, NM_000600), and *TNFα* (Hs_TNF_1_SG, NM_000594). The analysis was performed using the QIAquant 96 plex (Qiagen). Changes in mRNA expression levels were calculated using the comparative ΔΔCt method.

### Immunofluorescence

Fibroblasts were seeded on glass 8-well chamber slides (Corning, New York, NY) and treated for 24 h with 5μM ataluren or 25μM amlexanox. Slides were fixed with 4% paraformaldehyde for 15 min and rinsed five times with ice-cold PBS. Non-specific binding of antibodies was prevented by incubating samples with a 1% BSA, 0.1% Triton-X solution in PBS for one hour. Subsequently, cells were incubated overnight at +4 °C with rabbit monoclonal APC-conjugated anti-lamin B1 antibody (130-128-143, Miltenyi, Bergisch Gladbach, Germany, dilution 1:50), rabbit polyclonal anti-FANCC (ab97575, Abcam, dilution 1:150), or rabbit polyclonal anti-vimentin (GTX100619, Genetex, Irvine, CA, dilution 1:250). Slides were then washed and incubated with goat anti-rabbit FITC-conjugated antibody (F9887, Sigma-Aldrich, dilution 1:150) or goat anti-rabbit DyLight755-conjugated antibody (NBP2-60661IR, Novus Biologicals, Colorado, USA, dilution 1:1000). After three washes, samples were mounted on a coverslip using Pro-Long Gold antifade reagent with DAPI (ThermoFisher). Images were captured through a Zeiss Observer 7 inverted microscope (Zeiss, Oberkochen, Germany) and an Orca Flash 4.0 camera (Hamamatsu, Shizuoka, Japan), then analyzed by Zen software version 3.6 (Zeiss). Mean fluorescence intensity was calculated by ImageJ 1.54 g version (NIH, Bethesda, MD).

### MMC test

Mitomycin C (MMC) survival assays were performed according to a previously established standard method [26]. MMC protocol is reported in Supplementary Data 1.

### DEB test

The cytogenetics-based DEB (1,3 butadiene diepoxide, 202533, Sigma-Aldrich) test was performed on FA-A and FA-F LCL cultures as indicated in Supplementary Data 1.

### Statistical analysis

Independent groups were tested using the Mann-Whitney test, while the Wilcoxon signed-rank test or Student’s t-test were used in the case of paired data, depending on the Shapiro-Wilk normality test. For multiple group comparisons, Welch’s ANOVA test, mixed effect analysis or one-way ANOVA were employed. A *p*-value < 0.05 was considered statistically significant. The statistical software Prism version 8.0.1 (GraphPad, La Jolla, CA) was used.

## Supplementary information

Supplementary Data

Original Data

## Data Availability

All data generated or analyzed in this study are included in the published article and its supplementary information files.
