# Supplementary material for: Development of translational read-through-inducing drugs as novel therapeutic options for patients with Fanconi anemia
Source: Cell Death Discov. 2025 Jun 21;11:286. doi: 10.1038/s41420-025-02571-0 (PMC12182573; doi:10.1038/s41420-025-02571-0)
Supplement: Supplementary file 1 — Supplementary Data [file 41420_2025_2571_MOESM1_ESM.pdf]

Supplementary Material to:

Development of translational read-through-inducing drugs as novel therapeutic options for patients with Fanconi anemia

Correspondence to: Valentino Bezzerri, Cystic Fibrosis Center, Azienda Ospedaliera Universitaria Integrata, P.le A. Stefani 1, 37126, Verona, Italy. Email: v.bezzerri@unilink.it

Supplementary methods:

MMC test

Briefly, lymphoblast cells were incubated for 9 days with 5 μM ataluren or 24 h with 25 μM amlexanox, then collected and exposed to increasing concentrations of MMC (0–33 nM) for 5 days. Finally, cells were washed and resuspended in PBS plus 0.05% BSA and 0.5μg/ml propidium iodide, for 10 mins at 4°C. Cell viability was analyzed by flow cytometry.

DEB test

Cells were cultured for one, two or three weeks in the presence of ataluren (2.5 μM or 5 μM) or amlexanox (25 μM), replacing the media every two or three days with the addition of fresh drugs. Parallel untreated cultures were conducted as negative controls. At the end of each week, cultures were divided into two flasks with drugs conventionally added, as in the previous steps. The two divided flasks were treated with DEB at a final concentration of 0.1μg/ml for 72h. Negative cultures were treated in parallel, with only DEB, in the same way. At the end of the DEB treatment, colcemid solution (Euroclone, Rho, Italy) was added to each culture at a final concentration of 40 ng/ml and incubated for 2h. Cytogenetics specimens were prepared with standard hypotonic treatment followed by methanol/acetic acid treatment. Slides with metaphase spreads were stained with 10% Giemsa stain modified solution (32884, Sigma-Aldrich), after which a suitable number of metaphases were analyzed by DM5000B microscope (Leica, Nussloch, Germany) for the presence of chromosome or chromatide breakages, triradial figures, quadriradial figures or complex chromosomal figures.

Supplementary Table 1: Characteristics of patient-derived cell models employed in the study

ID	Cell type	FANC mut	Genotype	Protein mutation	Genetic engineering
SB-LCL	LCL	FANCA	c.2398G>T/c.3521G>A	p.Glu800x/p.Trp1174x	NA
SI-LCL	LCL	FANCA	c.2398G>T/c.3521G>A	p.Glu800x/p.Trp1174x	NA
SA-LCL	LCL	FANCA	c.2398G>T/c.3521G>A	p.Glu800x/p.Trp1174x	NA
EUFA121.L	LCL	FANCF	c.349_395del/c.16C>T	p.Gln6x	NA
OL-FCL	Primary fibroblasts	FANCA	p.Ala454Serfs*3	p.Glu345Valfs*63	NA
OL-FCL-Corr	Primary fibroblasts	FANCA	Wild type	Wild type	FANCA correction
AL-FCL (FA-C)	Primary fibroblasts	FANCC	c.1642C>T/c. 1642C>T	p.Arg548x/ p.Arg548x	NA

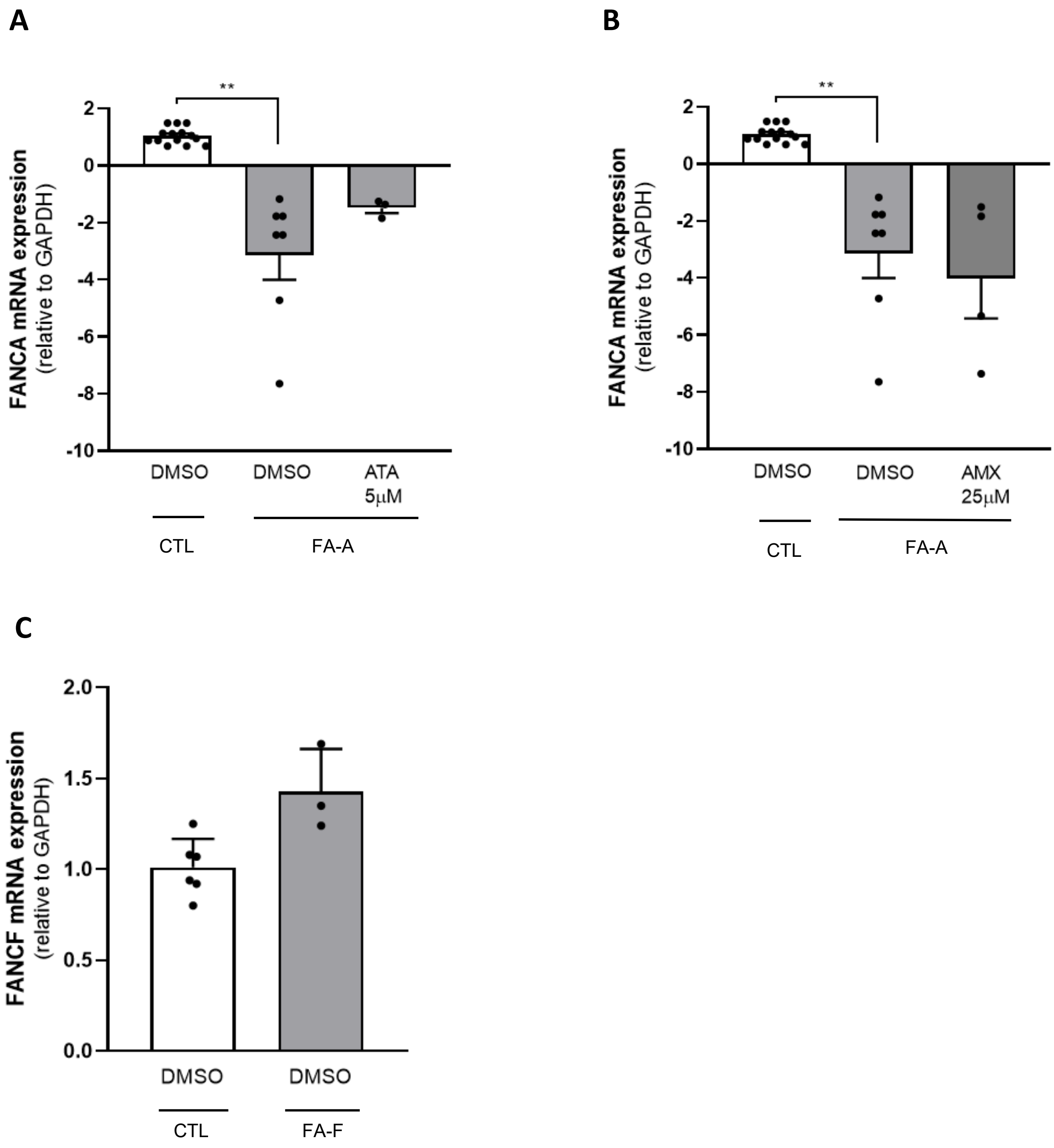

Supplementary Figure 1: Nonsense-mutated *FANCA* mRNA undergoes significant nonsense-mediated mRNA decay (NMD) that is slightly reduced with ataluren but not amlexanox, while nonsense-mutated *FANCF* mRNA does not undergo NMD. mRNA expression levels of *FANCA* in FA-A mutated LCL and after incubation with ataluren 5μM (**A**, n=3) and amlexanox 25μM (**B**, n=4). mRNA expression levels of *FANCF* in FA-F mutated LCL (**C**, n=3). Data are represented as mean ± SEM. Statistical significance was assessed using paired t-test (**p<0.01).

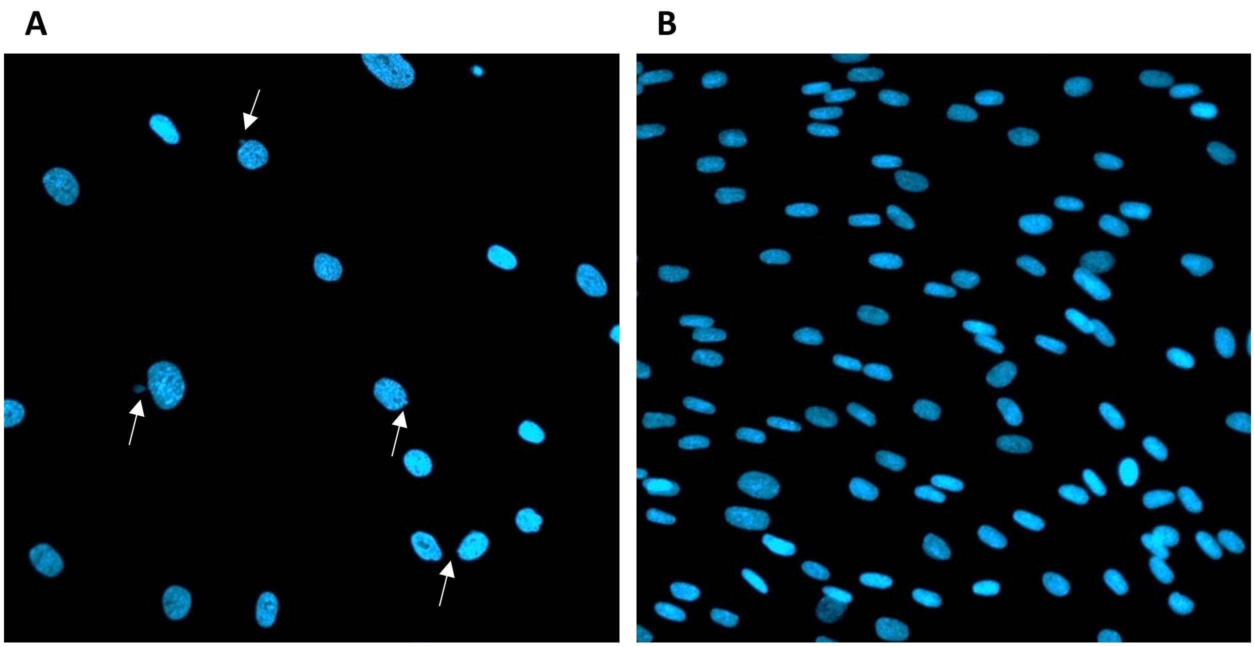

Supplementary Figure 2: *FANCA*-mutat fibroblasts exhibit frequent micronuclei compared to their isogenic corrected cells. Immunofluorescence staining for DAPI in isogenic *FANCA*-mutant (OL-FCL) (**A**) and corrected (OL-FCL-Corr) (**B**) fibroblasts. All pictures are taken at 20x magnification. Micronuclei are indicated with white arrows.

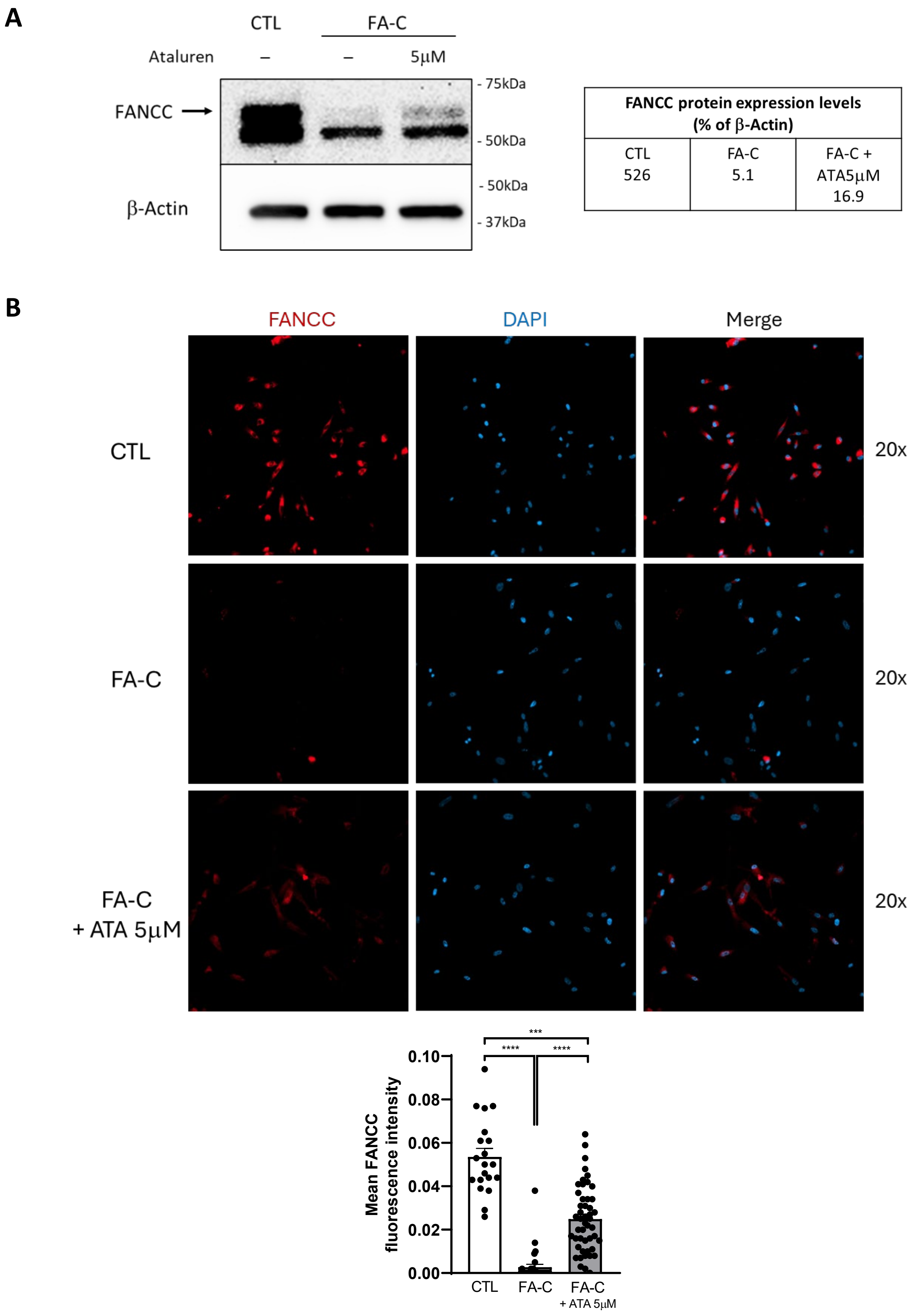

Supplementary Figure 3: Ataluren restores full-length FANCC protein expression in *FANCC* nonsense-mutated primary fibroblasts. FANCC Western blot analysis (**A**) and immunofluorescence staining (**B,** n=48), with their respective quantifications, of primary *FANCC* nonsense-mutated fibroblasts (FA-C) before and after

24h incubation with ataluren 5 μM, compared to healthy controls (CTL). All immunofluorescence images were taken at 20x magnification. Data are represented as mean ± SEM; statistical significance was calculated using Kruskal-Wallis test (***$p<0.001$; ****$p<0.0001$).

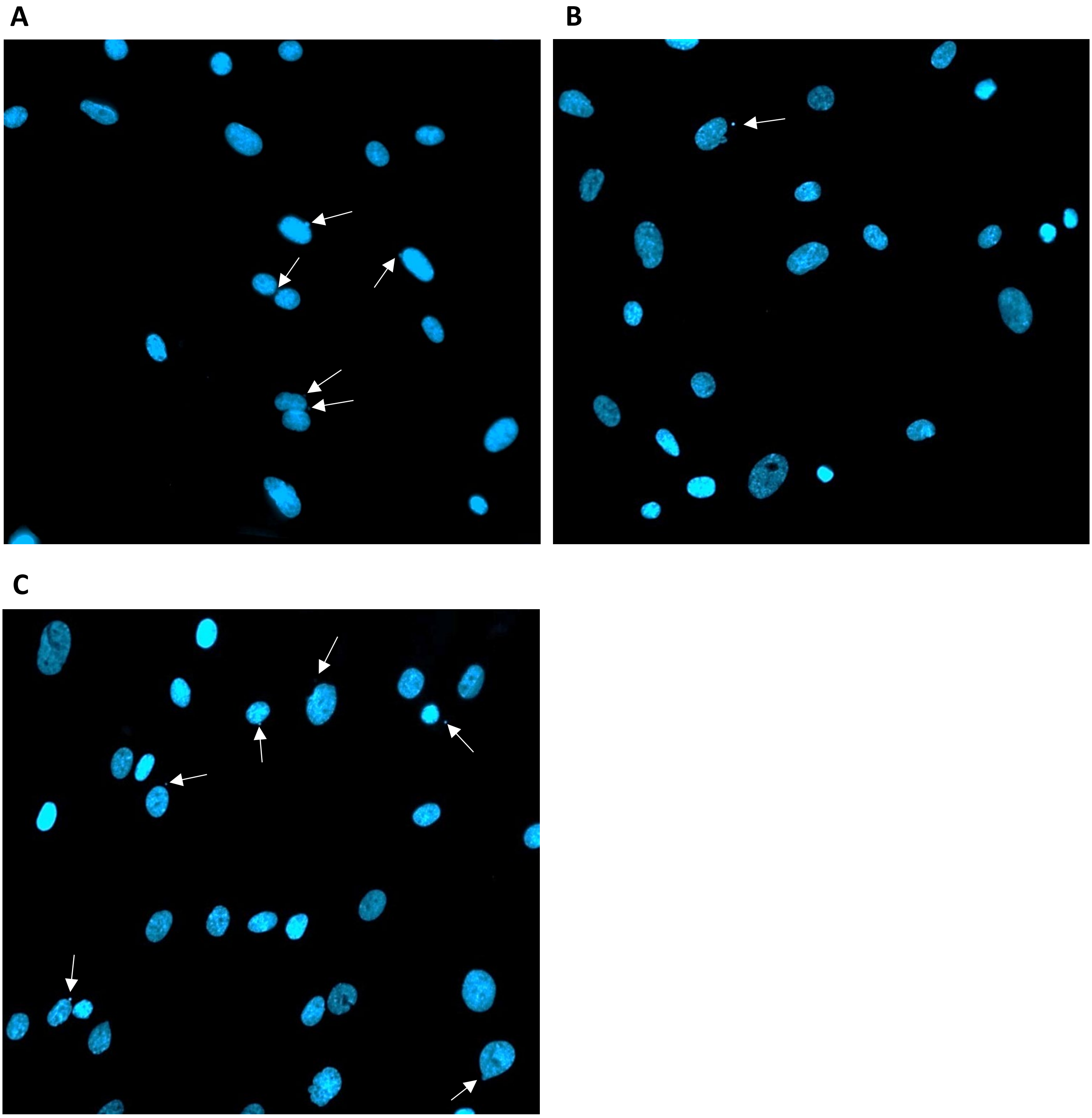

Supplementary Figure 4: *FANCC*-mutated primary fibroblasts exhibit numerous micronuclei that are reduced after incubation with ataluren. Immunofluorescence staining for DAPI of nonsense *FANCC*-mutant fibroblasts (**A**) incubated for 24h with ataluren 5μM (**B**) and amlexanox 25μM (**C**). All images were acquired at 20x magnification. Micronuclei are indicated with white arrows.
