# Supplementary material for: Development of translational read-through-inducing drugs as novel therapeutic options for patients with Fanconi anemia
Source: Cell Death Discov. 2025 Jun 21;11:286. doi: 10.1038/s41420-025-02571-0 (PMC12182573; doi:10.1038/s41420-025-02571-0)

Original data to:

Development of translational read-through-inducing drugs as novel therapeutic options for patients with Fanconi anemia

<u>Figure 1</u>

1.A:

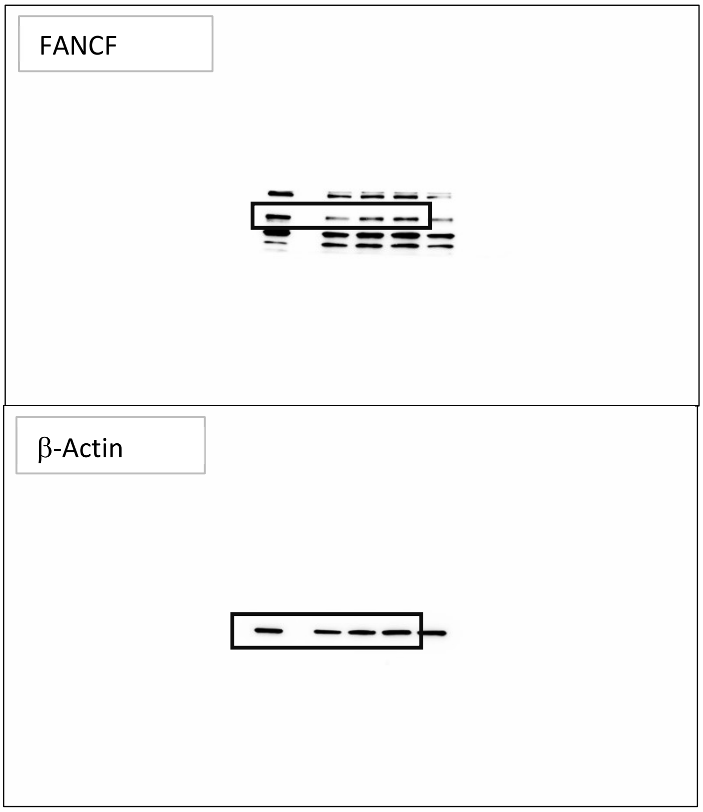

1.B

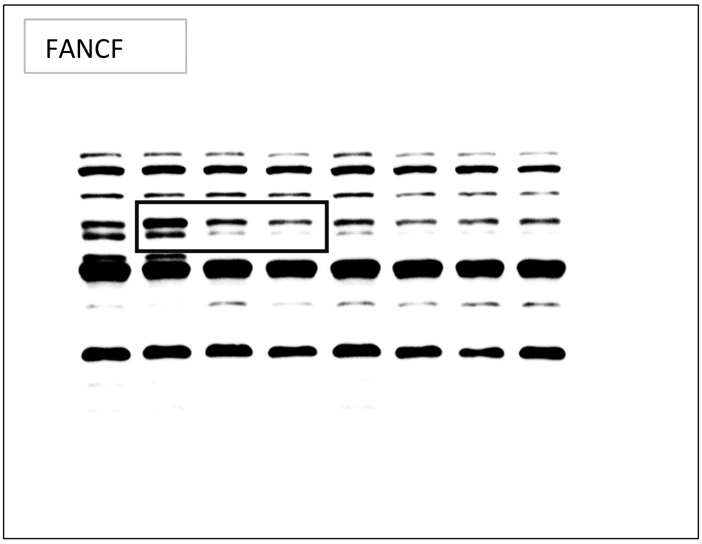

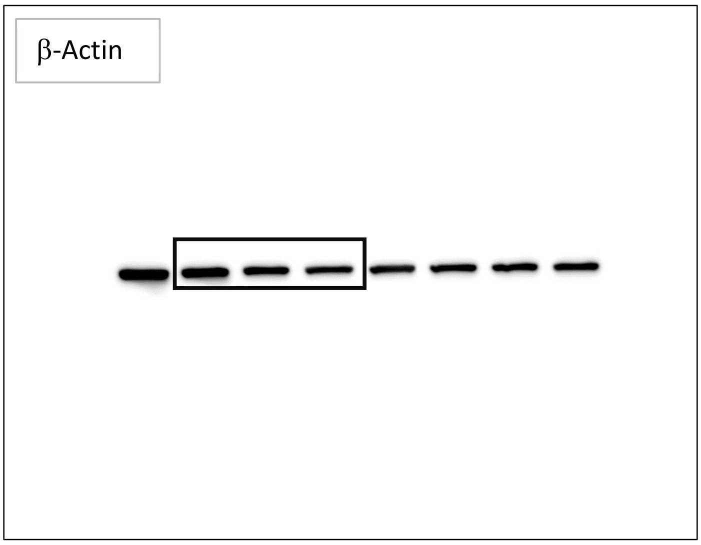

1.C

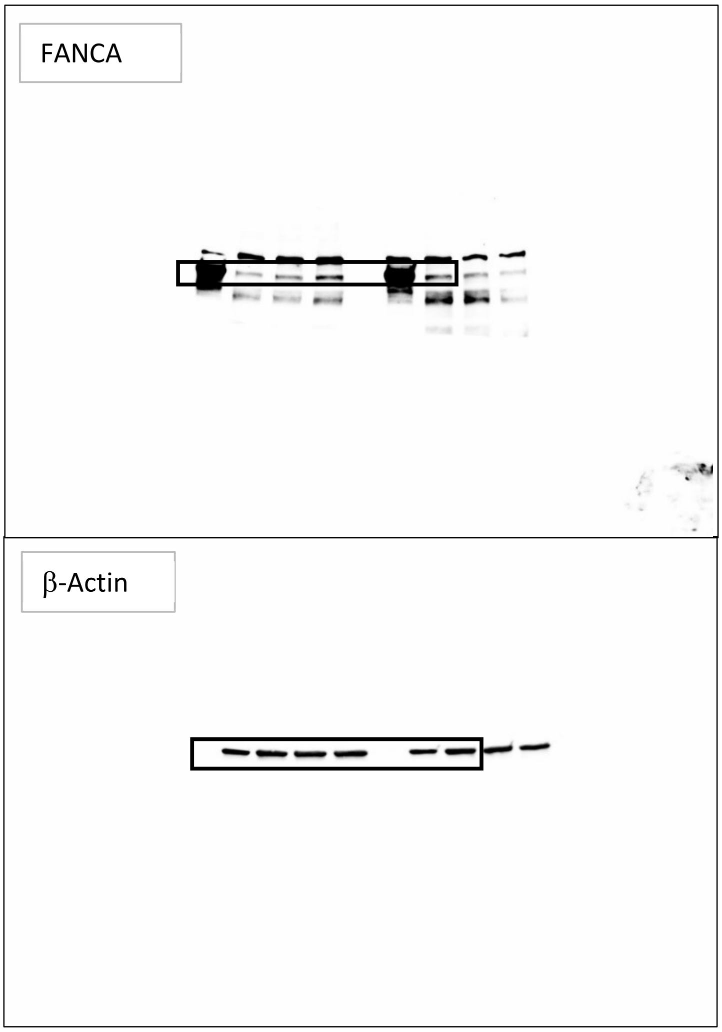

Figure 2

2.A

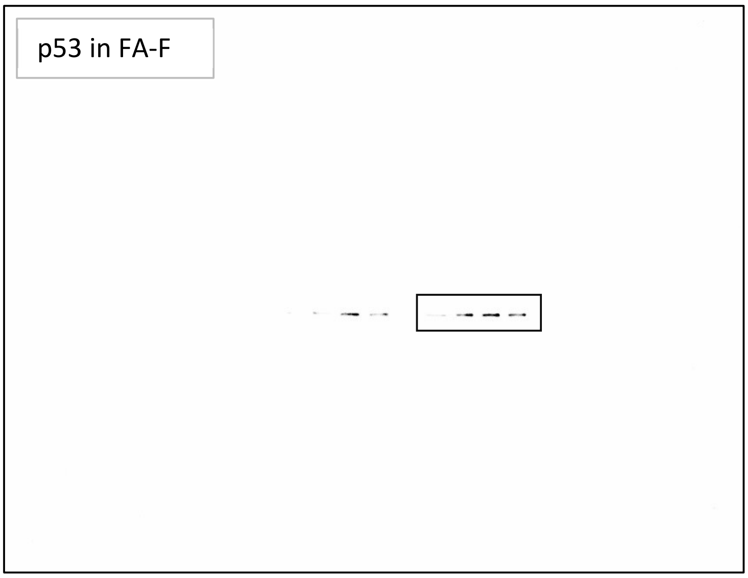

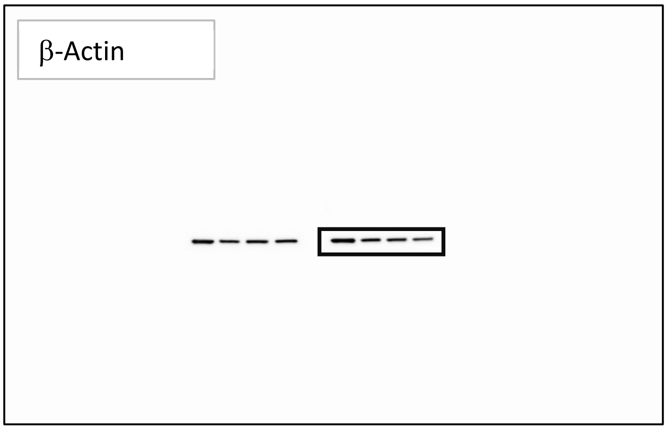

2.B

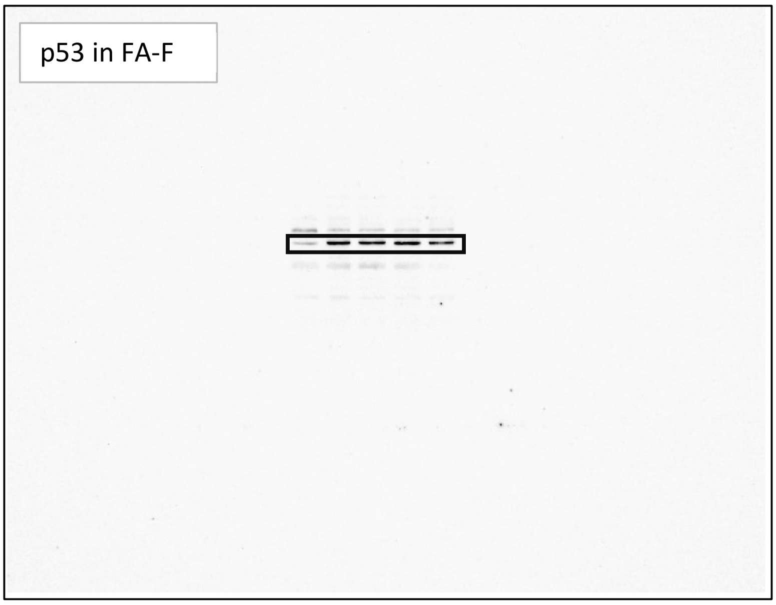

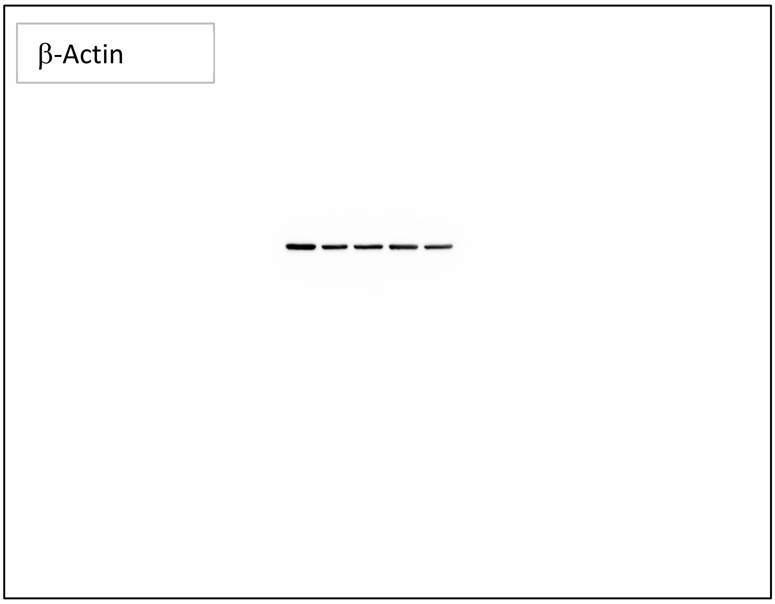

2.C

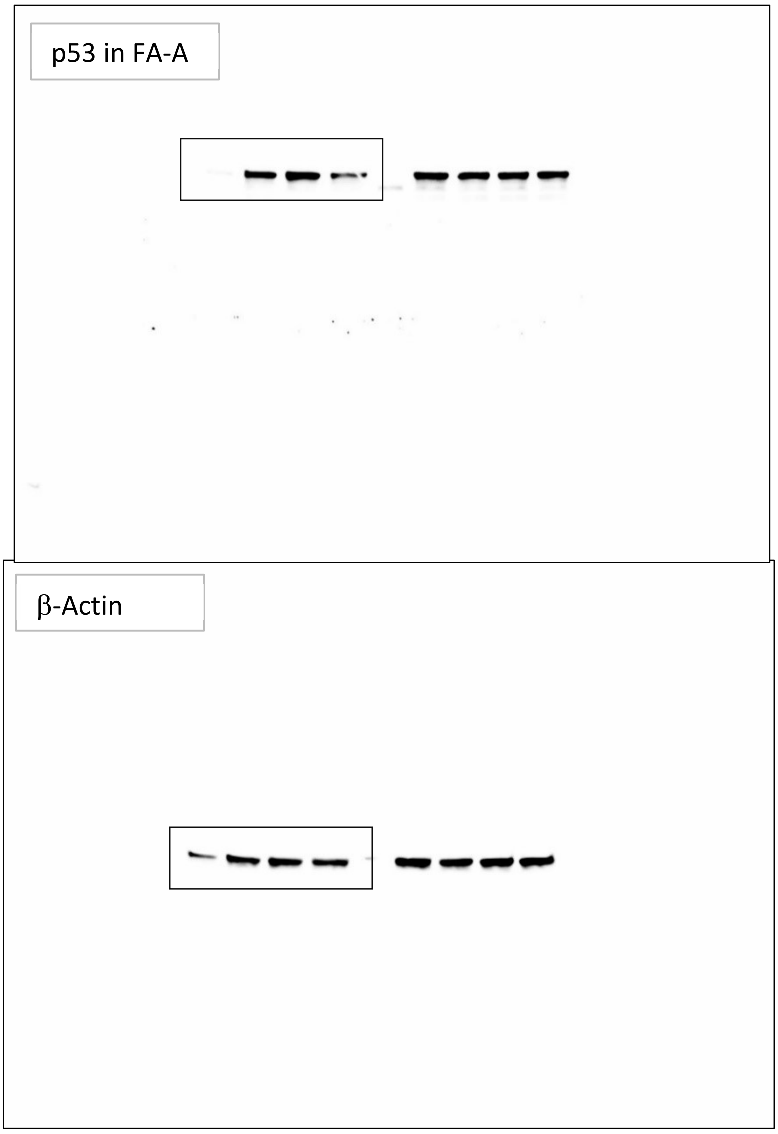

2.D

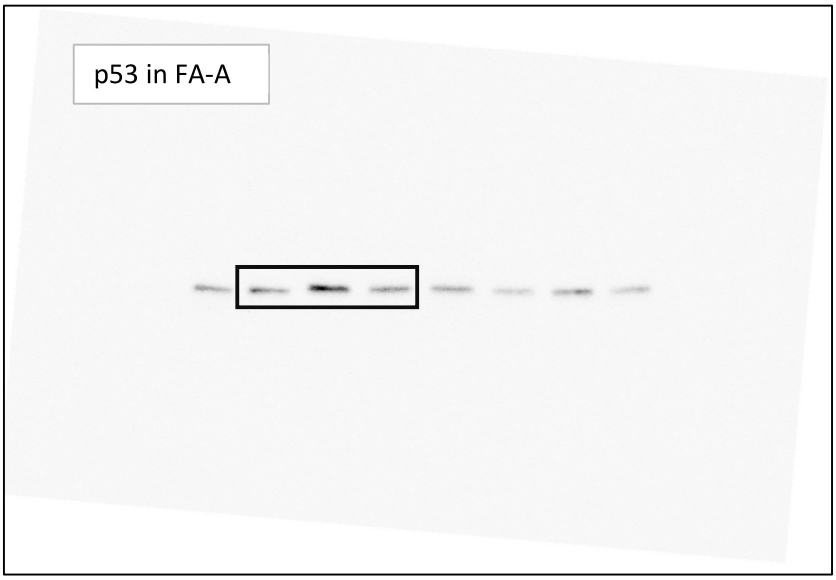

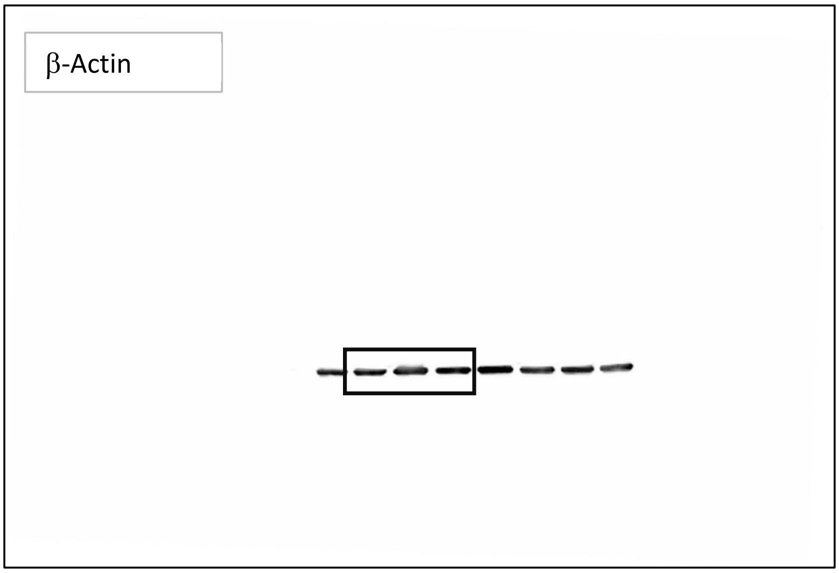

Fig 3

3.A

MMC (uM)	CTL (cell viability, %)				FA-F (cell viability, %)				FA-F + ATA 5uM (cell, viability, %)			
0	100	100	100	100	100	100	100	100	100	100	100	100
3	98,5	95	98	96	59	58	62	57	91	84	77	84
10	100	91	93	92	34	35	38	49	72	76	49	61
33	87,5	79	88	78	17	18	20	25	55	31	35	47

3.B

MMC (uM)	CTL (cell viability, %)				FA-A (cell viability, %)							FA-A + ATA 5uM (cell viability, %)						
0	100	100	100	100	100	100	100	100	100	100	100	100	100	100	100	100	100	100
3	98,5	95	98	96	55,8	73,2	64	73	66	66	59	62,1	83,3	73	83	72	81	78
10	100	91	93	92	44,1	40,8	48		50	62	53	54,1	61,7	64	N/A	63	75	70
33	87,5	79	88	78	33,8	28,2	40	41	35	31	36	35,1	38,3	51	62	45	55	55

3.C

**DEB Test FA-F (Ata 2.5uM, 5uM)**								
Treatment		Mytosis (n)	Cromatid Breaks	Chromosome Breaks	Triradial figures	Quadriradial figures	Complex	Breakage/Mytosis
DEB (positive control)		58	112	25	18	1	0	3,02
Negative control (NO DEB)		20	2	0	0	0	0	0,1
Ataluren 2,5µM		20	13	16	5	0	4	2,35
parallel treatment Ata + DEB								
Ataluren 5µM		20	29	5	9	0	1	2,7
Parallel treatment Ata + DEB								
Ataluren 2,5µM		29	10	0	0	0	0	0,34
1 week treatment + DEB								
Ataluren 5µM		90	11	0	0	0	0	0,12
1 week treatment + DEB								
Ataluren 2,5µM		-	-	-	-	-	-	
2 weeks treatment + DEB								
Ataluren 5µM		50	8	0	0	0	0	0,16
2 weeks treatment + DEB								
Ataluren 2,5µM		-	-	-	-	-	-	
3 weeks treatment + DEB								
Ataluren 5µM		20	0	0	0	0	0	0
3 weeks treatment + DEB								

3.D

**DEB Test FA-A (Ata 2.5uM, 5uM)**								
Treatment		Mytosis	Cromatid Breaks	Chromosome Breaks	Triradial figure	Quadriradial Figures	Complex	Breakage/Mytosis
DEB (positive control)		50	80	5	7	-	-	1,98
Negative control		50	7	0	0	0	0	0,14
Ataluren 2,5µM		30	12	0	2	1	0	0,6
1 week treatment + DEB								
Ataluren 5µM		20	2	0	0	0	0	0,1
1 week treatment + DEB								
Ataluren 2,5µM		27	0	0	0	0	0	0
2 weeks treatment + DEB								
Ataluren 5µM		50	0	0	0	0	0	0
2 weeks treatment + DEB								
Ataluren 2,5µM		50	4	0	0	0	0	0,08
3 weeks treatment + DEB								
Ataluren 5µM		50	2	0	1	0	0	0,08
3 weeks treatment + DEB								

Figure 4

4.A

MMC (uM)	CTL (cell viability, %)				FA-F (cell viability, %)			FA-F + AMX 25uM (cell viability, %		
0	100	100	100	100	100	100	100	100	100	100
3	98,5	95	98	96	73	74,14	82,61	73,6	54,72	77,05
10	100	91	93	92	66,4	60,34	88,41	68,2	56,6	57,38
33	87,5	79	88	78	60,8	53,45	53,62	66,1	26,42	47,54

4.B

MMC (uM)	CTL (cell viability, %)				FA-A (cell viability, %)			FA-A + AMX 25uM (cell viability, %		
0	100	100	100	100	100	100	100	100	100	100
3	98,5	95	98	96	57,14	73,21	46,55	50	64,81	46,15
10	100	91	93	92	46,43	58,93	34,48	36,36	46,3	30,77
33	87,5	79	88	78	32,14	41,07	25,86	22,73	35,19	21,15

4.C

**DEB Test FA-A (AMX25uM)**							
Treatment	Mytosis (n)	Cromatid Breaks	Chromosome Breaks	Triradial figures	Quadriradial Figures	Complex	Breakage/Mytosis
DEB (positive control)	21	25	6	5	0	0	1,95
Negative control (NO DEB)	30	2	0	0	0	0	0,07
Amlexanox 25µM	15	11	4	9	1	0	2,33
1 week treatment + DEB							
Amlexanox 25µM	17	17	7	3	3	0	2,12
2 weeks treatment + DEB							
Amlexanox 25µM	40	41	13	4	0	0	1,55
3 weeks treatment + DEB							

Figure 5

5.A

	Exp #1						Exp #2					
IL6	36,60	32,79	33,18	33,18	33,63	32,36	38,40	33,21	33,44	33,01	34,29	33,37
	36,42	32,88	33,03	32,98	33,46	32,22	37,04	32,73	33,29	32,89	34,09	33,26
sum	73,02	65,67	66,21	66,16	67,09	64,58	75,44	65,94	66,73	65,90	68,38	66,63
mean	36,51	32,84	33,11	33,08	33,55	32,29	37,72	32,97	33,37	32,95	34,19	33,32
st dev	0,13	0,06	0,11	0,14	0,12	0,10	0,96	0,34	0,11	0,08	0,14	0,08
GAPDH	18,17	18,42	18,45	18,66	18,65	18,77	18,36	18,72	18,63	18,67	18,82	19,21
	18,10	18,64	18,26	18,38	18,58	18,64	18,47	18,66	18,44	18,41	18,83	19,04
sum	36,27	37,06	36,71	37,04	37,23	37,41	36,83	37,38	37,07	37,08	37,65	38,25
mean	18,14	18,53	18,36	18,52	18,62	18,71	18,42	18,69	18,54	18,54	18,83	19,13
st dev	0,05	0,16	0,13	0,20	0,05	0,09	0,08	0,04	0,13	0,18	0,01	0,12
	**CTL NT**	**FA-A NT**	**FA-A+AM**	**FA-A+PT**	**FA-A+HC**	**FA-A+NFkBin**	**CTL NT**	**FA-A NT**	**FA-A+AM**	**FA-A+PT**	**FA-A+HC**	**FA-A+NFkBin**
DCt	18,38	14,31	14,75	14,56	14,93	13,59	19,31	14,28	14,83	14,41	15,37	14,19
	0,02	0,03	0,03	0,06	0,02	0,02	0,93	0,12	0,03	0,04	0,02	0,02
st dev	0,14	0,17	0,17	0,24	0,13	0,14	0,96	0,34	0,17	0,20	0,14	0,14
dct	18,38	14,31	14,75	14,56	14,93	13,59	19,31	14,28	14,83	14,41	15,37	14,19
	0,05	0,16	0,13	0,20	0,05	0,09	0,08	0,04	0,13	0,18	0,01	0,12
**calibrator**	18,38	18,38	18,38	18,38	18,38	18,38	19,31	19,31	19,31	19,31	19,31	19,31
st dev	0,05	0,05	0,05	0,05	0,05	0,05	0,08	0,08	0,08	0,08	0,08	0,08
ddCT	0,00	-4,08	-3,63	-3,82	-3,45	-4,80	0,00	-5,03	-4,48	-4,90	-3,95	-5,12
	0,00	0,03	0,02	0,04	0,00	0,01	0,01	0,01	0,02	0,04	0,01	0,02
	0,07	0,16	0,14	0,20	0,07	0,10	0,11	0,09	0,16	0,20	0,08	0,14
	-0,08	-4,24	-3,77	-4,02	-3,52	-4,90	-0,12	-5,12	-4,64	-5,10	-4,03	-5,26
	0,07	-3,91	-3,49	-3,62	-3,38	-4,69	0,11	-4,94	-4,32	-4,70	-3,86	-4,98
max	1,05	18,87	13,67	16,27	11,47	29,85	1,08	34,79	24,87	34,31	16,28	38,44
Expression	**1,00**	**16,85**	**12,38**	**14,12**	**10,93**	**27,76**	**1,00**	**32,67**	**22,32**	**29,86**	**15,40**	**34,78**
min	0,96	15,05	11,21	12,26	10,41	25,82	0,93	30,68	20,02	25,98	14,57	31,46
	0,97	0,97	0,97	0,97	0,97	0,97	0,95	0,95	0,95	0,95	0,95	0,95
	1,04	1,04	1,04	1,04	1,04	1,04	1,06	1,06	1,06	1,06	1,06	1,06

	Exp #3											
IL6	34,67	31,58	32,88	32,55	33,30	31,84	36,22	33,04	34,72	33,50	33,86	33,48
	34,73	31,52	32,95	32,99	33,48	31,80	35,59	33,02	35,10	33,44	33,49	32,77
sum	69,40	63,10	65,83	65,54	66,78	63,64	71,81	66,06	69,82	66,94	67,35	66,25
mean	34,70	31,55	32,92	32,77	33,39	31,82	35,91	33,03	34,91	33,47	33,68	33,13
st dev	0,04	0,04	0,05	0,31	0,13	0,03	0,45	0,01	0,27	0,04	0,26	0,50
GAPDH	17,79	18,54	18,79	19,04	19,49	18,45	18,18	17,82	18,04	17,70	17,94	17,07
	18,06	18,44	19,04	18,95	19,49	18,50	18,03	18,02	17,82	17,75	18,06	17,01
sum	35,85	36,98	37,83	37,99	38,98	36,95	36,21	35,84	35,86	35,45	36,00	34,08
mean	17,93	18,49	18,92	19,00	19,49	18,48	18,11	17,92	17,93	17,73	18,00	17,04
st dev	0,19	0,07	0,18	0,06	0,00	0,04	0,11	0,14	0,16	0,04	0,08	0,04
	**CTL NT**	**FA-A NT**	**FA-A+AM**	**FA-A+PT**	**FA-A+HC**	**FA-A+NFkBin**	**CTL NT**	**FA-A NT**	**FA-A+AM**	**FA-A+PT**	**FA-A+HC**	**FA-A+NFk**
DCt	16,78	13,06	14,00	13,78	13,90	13,35	17,80	15,11	16,98	15,75	15,68	16,09
	0,04	0,01	0,03	0,10	0,02	0,00	0,21	0,02	0,10	0,00	0,08	0,25
st dev	0,20	0,08	0,18	0,32	0,13	0,05	0,46	0,14	0,31	0,06	0,28	0,50
dct	16,78	13,06	14,00	13,78	13,90	13,35	17,80	15,11	16,98	15,75	15,68	16,09
	0,19	0,07	0,18	0,06	0,00	0,04	0,11	0,14	0,16	0,04	0,08	0,04
**calibrator**	16,78	16,78	16,78	16,78	16,78	16,78	17,8	17,8	17,8	17,8	17,8	17,8
st dev	0,19	0,19	0,19	0,19	0,19	0,19	0,11	0,11	0,11	0,11	0,11	0,11
ddCT	0,00	-3,72	-2,78	-3,01	-2,88	-3,44	0,00	-2,69	-0,82	-2,06	-2,13	-1,72
	0,07	0,04	0,07	0,04	0,04	0,04	0,02	0,03	0,04	0,01	0,02	0,01
	0,27	0,20	0,26	0,20	0,19	0,19	0,15	0,18	0,19	0,12	0,14	0,12
	-0,27	-3,92	-3,04	-3,21	-3,07	-3,63	-0,15	-2,87	-1,01	-2,17	-2,26	-1,83
	0,26	-3,52	-2,52	-2,80	-2,69	-3,24	0,15	-2,51	-0,63	-1,94	-1,99	-1,60
max	1,21	15,17	8,22	9,22	8,40	12,37	1,11	7,31	2,01	4,50	4,80	3,56
Expression	**1,00**	**13,18**	**6,87**	**8,03**	**7,36**	**10,82**	**1,00**	**6,45**	**1,77**	**4,16**	**4,36**	**3,28**
min	0,83	11,45	5,74	6,99	6,45	9,46	0,90	5,70	1,55	3,84	3,96	3,03
	0,88	0,88	0,88	0,88	0,88	0,88	0,93	0,93	0,93	0,93	0,93	0,93
	1,14	1,14	1,14	1,14	1,14	1,14	1,08	1,08	1,08	1,08	1,08	1,08

5.B

	Exp #1						Exp #2					
TNFa	24,21	23,17	22,91	23,54	24,48	23,02	23,95	23,25	22,71	22,72	24,70	23,09
	24,10	23,17	23,04	23,56	24,55	22,79	23,93	23,07	22,93	22,76	24,69	23,09
sum	48,31	46,34	45,95	47,10	49,03	45,81	47,88	46,32	45,64	45,48	49,39	46,18
mean	24,16	23,17	22,98	23,55	24,52	22,91	23,94	23,16	22,82	22,74	24,70	23,09
st dev	0,08	0,00	0,09	0,01	0,05	0,16	0,01	0,13	0,16	0,03	0,01	0,00
GAPDH	18,51	18,72	18,74	18,82	18,73	18,92	18,10	18,20	17,87	17,97	18,34	18,59
	18,43	18,64	18,43	18,81	18,50	18,72	18,20	18,12	17,88	17,83	18,31	18,54
sum	36,94	37,36	37,17	37,63	37,23	37,64	36,30	36,32	35,75	35,80	36,65	37,13
mean	18,47	18,68	18,59	18,82	18,62	18,82	18,15	18,16	17,88	17,90	18,33	18,57
st dev	0,06	0,06	0,22	0,01	0,16	0,14	0,07	0,06	0,01	0,10	0,02	0,04
	**CTL NT**	**FA-A NT**	**FA-A+AM**	**FA-A+PT**	**FA-A+HC**	**FA-A+NFkBin**	**CTL NT**	**FA-A NT**	**FA-A+AM**	**FA-A+PT**	**FA-A+HC**	**FA-A+NFkBin**
DCt	5,69	4,49	4,39	4,74	5,90	4,09	5,79	5,00	4,95	4,84	6,37	4,53
	0,01	0,00	0,06	0,00	0,03	0,05	0,01	0,02	0,02	0,01	0,00	0,00
st dev	0,10	0,06	0,24	0,02	0,17	0,22	0,07	0,14	0,16	0,10	0,02	0,04
dct	5,69	4,49	4,39	4,74	5,90	4,09	5,79	5,00	4,95	4,84	6,37	4,53
	0,06	0,06	0,22	0,01	0,16	0,14	0,07	0,06	0,01	0,10	0,02	0,04
**calibrator**	5,69	5,69	5,69	5,69	5,69	5,69	5,79	5,79	5,79	5,79	5,79	5,79
st dev	0,1	0,1	0,1	0,1	0,1	0,1	0,07	0,07	0,07	0,07	0,07	0,07
ddCT	0,00	-1,20	-1,30	-0,96	0,21	-1,61	0,00	-0,79	-0,85	-0,95	0,58	-1,27
	0,01	0,01	0,06	0,01	0,04	0,03	0,01	0,01	0,00	0,01	0,01	0,01
	0,11	0,11	0,24	0,10	0,19	0,17	0,10	0,09	0,07	0,12	0,07	0,08
	-0,12	-1,31	-1,54	-1,06	0,02	-1,78	-0,10	-0,88	-0,92	-1,07	0,51	-1,34
	0,11	-1,09	-1,06	-0,85	0,40	-1,43	0,10	-0,70	-0,77	-0,83	0,65	-1,19
max	1,09	2,49	2,91	2,08	0,99	3,43	1,07	1,84	1,89	2,10	0,70	2,54
Expression	**1,00**	**2,30**	**2,46**	**1,94**	**0,86**	**3,04**	**1,00**	**1,73**	**1,80**	**1,93**	**0,67**	**2,40**
min	0,93	2,12	2,08	1,81	0,76	2,70	0,93	1,62	1,71	1,78	0,64	2,28
	0,93	0,93	0,93	0,93	0,93	0,93	0,95	0,95	0,95	0,95	0,95	0,95
	1,07	1,07	1,07	1,07	1,07	1,07	1,05	1,05	1,05	1,05	1,05	1,05
					**-1,16**						**-1,49**	

	Exp #3											
TNFa	23,74	23,85	24,11	24,20	26,12	23,50	24,17	22,76	23,45	23,50	24,04	23,44
	23,70	23,87	24,01	24,31	26,38	23,73	24,23	22,80	23,34	23,49	24,16	23,43
sum	47,44	47,72	48,12	48,51	52,50	47,23	48,40	45,56	46,79	46,99	48,20	46,87
mean	23,72	23,86	24,06	24,26	26,25	23,62	24,20	22,78	23,40	23,50	24,10	23,44
st dev	0,03	0,01	0,07	0,08	0,18	0,16	0,04	0,03	0,08	0,01	0,08	0,01
GAPDH	18,03	18,13	18,90	18,67	19,32	18,67	18,18	17,82	18,04	17,70	17,94	17,07
	17,87	18,17	18,50	18,55	19,22	18,47	18,03	18,02	17,82	17,75	18,06	17,01
sum	35,90	36,30	37,40	37,22	38,54	37,14	36,21	35,84	35,86	35,45	36,00	34,08
mean	17,95	18,15	18,70	18,61	19,27	18,57	18,11	17,92	17,93	17,73	18,00	17,04
st dev	0,11	0,03	0,28	0,08	0,07	0,14	0,11	0,14	0,16	0,04	0,08	0,04
	**CTL NT**	**FA-A NT**	**FA-A+AM**	**FA-A+PT**	**FA-A+HC**	**FA-A+NFkBin**	**CTL NT**	**FA-A NT**	**FA-A+AM**	**FA-A+PT**	**FA-A+HC**	**FA-A+NFk**
DCt	5,77	5,71	5,36	5,65	6,98	5,05	6,10	4,86	5,47	5,77	6,10	6,40
	0,01	0,00	0,08	0,01	0,04	0,05	0,01	0,02	0,03	0,00	0,01	0,00
st dev	0,12	0,03	0,29	0,12	0,20	0,22	0,11	0,14	0,17	0,04	0,12	0,04
dct	5,77	5,71	5,36	5,65	6,98	5,05	6,10	4,86	5,47	5,77	6,10	6,40
	0,11	0,03	0,28	0,08	0,07	0,14	0,11	0,14	0,16	0,04	0,08	0,04
**calibrator**	5,77	5,77	5,77	5,77	5,77	5,77	6,1	6,1	6,1	6,1	6,1	6,1
st dev	0,12	0,12	0,12	0,12	0,12	0,12	0,11	0,11	0,11	0,11	0,11	0,11
ddCT	0,00	-0,06	-0,41	-0,13	1,21	-0,72	0,00	-1,24	-0,64	-0,33	0,00	0,30
	0,03	0,02	0,09	0,02	0,02	0,03	0,02	0,03	0,04	0,01	0,02	0,01
	0,16	0,12	0,31	0,15	0,14	0,19	0,15	0,18	0,19	0,12	0,14	0,12
	-0,16	-0,18	-0,72	-0,27	1,07	-0,91	-0,16	-1,42	-0,83	-0,45	-0,14	0,18
	0,16	0,06	-0,10	0,02	1,35	-0,54	0,15	-1,06	-0,44	-0,21	0,14	0,41
max	1,12	1,14	1,64	1,21	0,48	1,88	1,12	2,67	1,77	1,36	1,10	0,88
Expression	**1,00**	**1,04**	**1,33**	**1,09**	**0,43**	**1,65**	**1,00**	**2,36**	**1,55**	**1,26**	**1,00**	**0,82**
min	0,89	0,96	1,07	0,98	0,39	1,45	0,90	2,09	1,36	1,16	0,91	0,75
	0,92	0,92	0,92	0,92	0,92	0,92	0,93	0,93	0,93	0,93	0,93	0,93
	1,09	1,09	1,09	1,09	1,09	1,09	1,08	1,08	1,08	1,08	1,08	1,08
					**-2,31**							**-1,23**

5.C

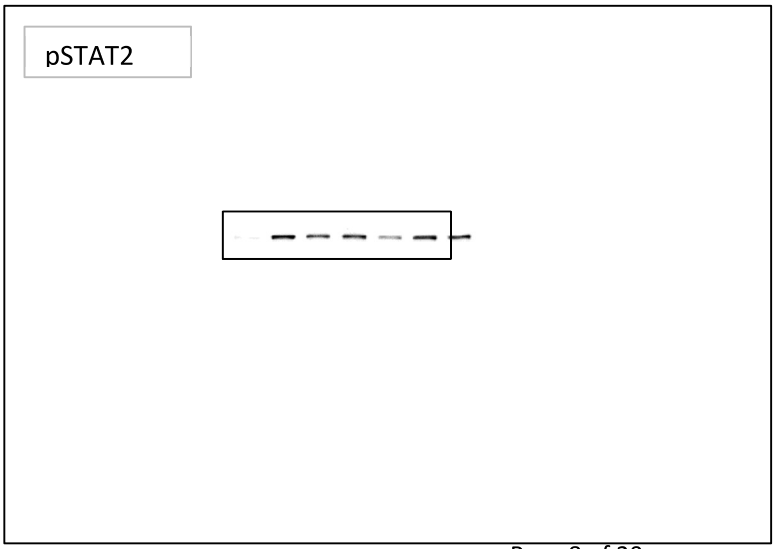

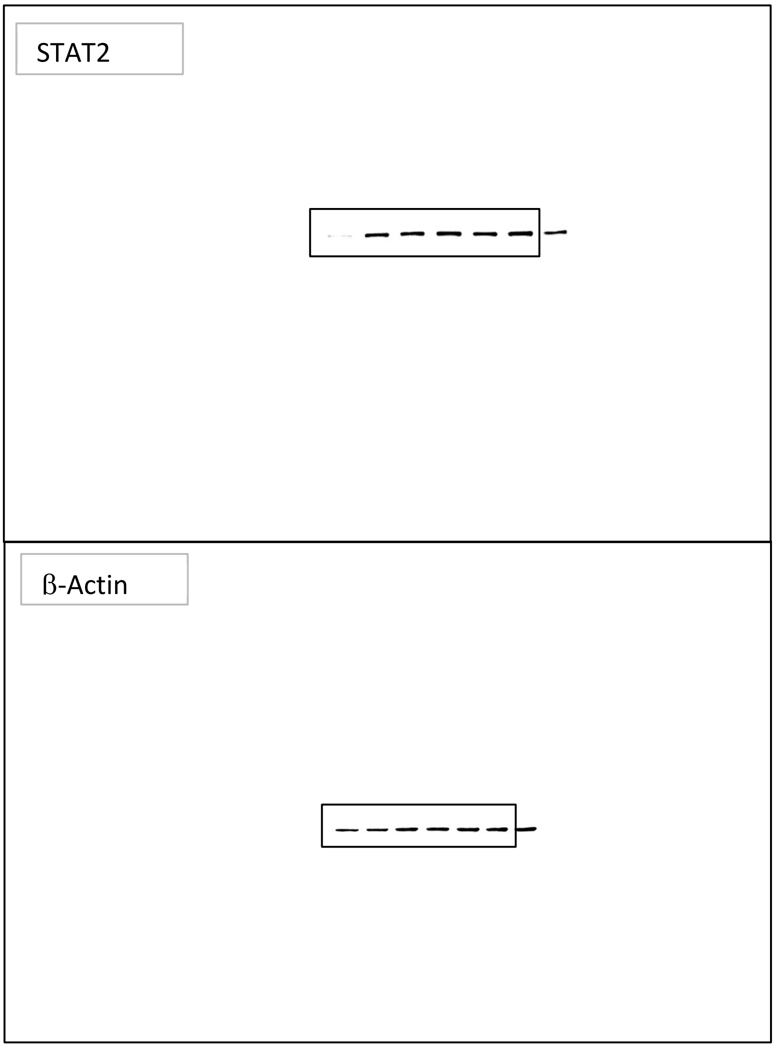

5.D

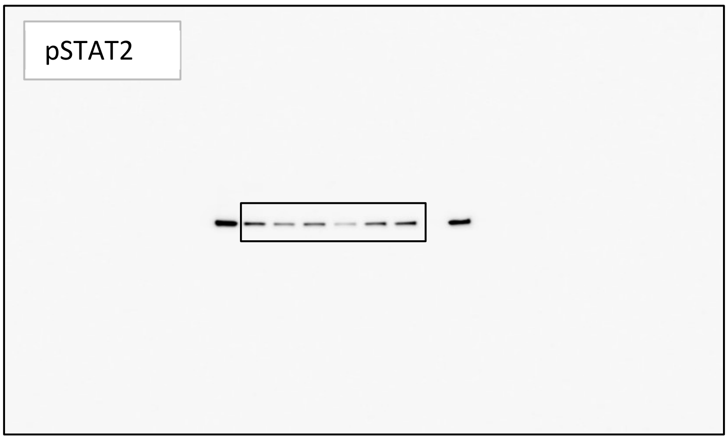

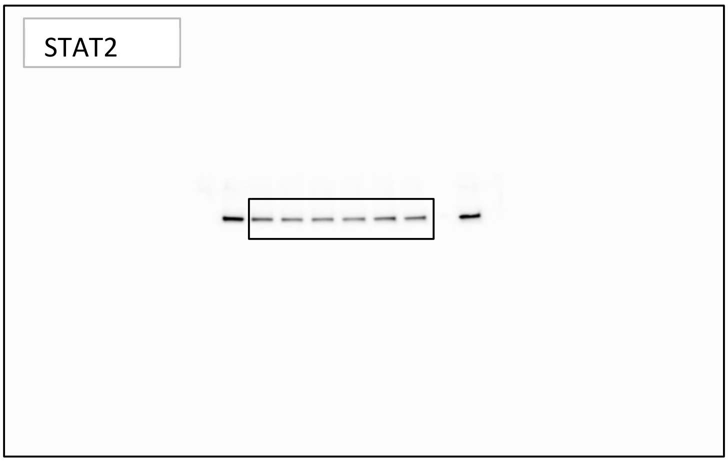

5.E

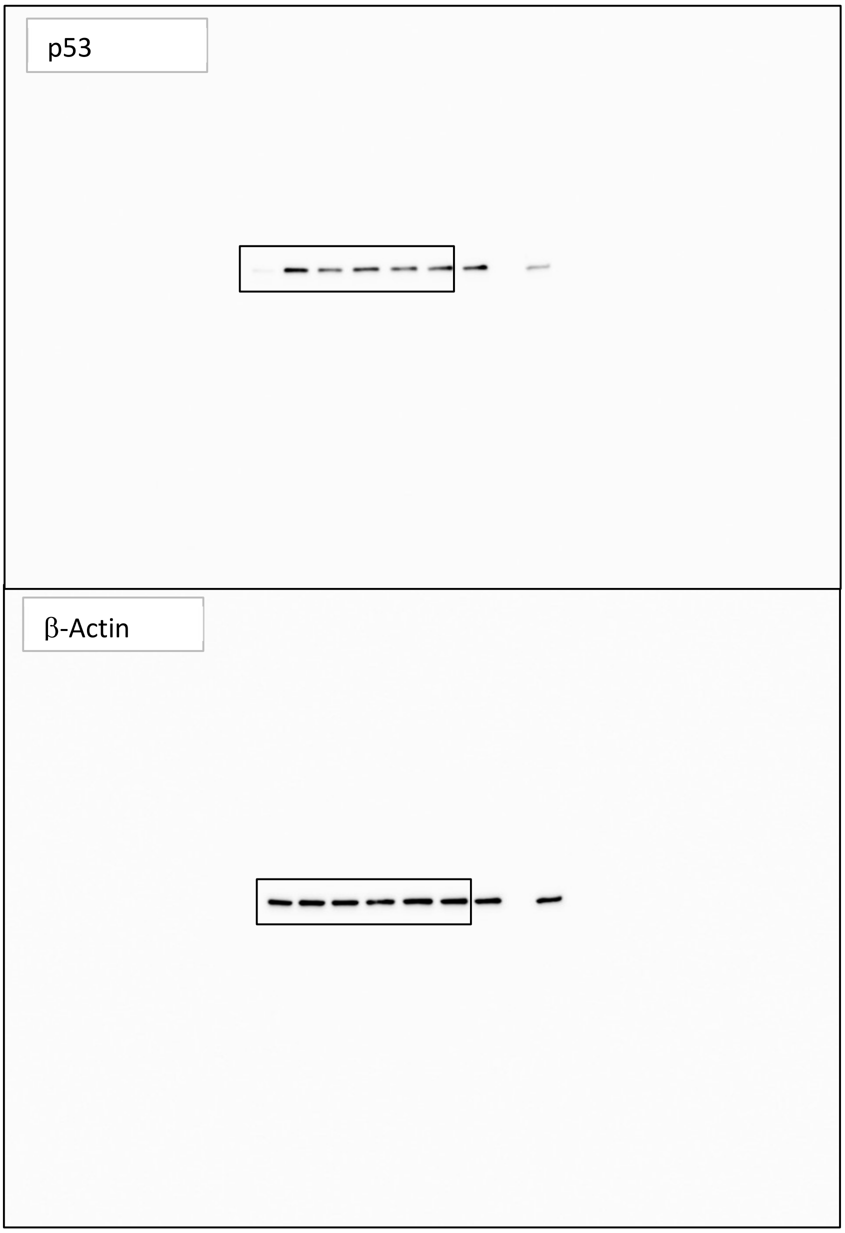

Fig. 6

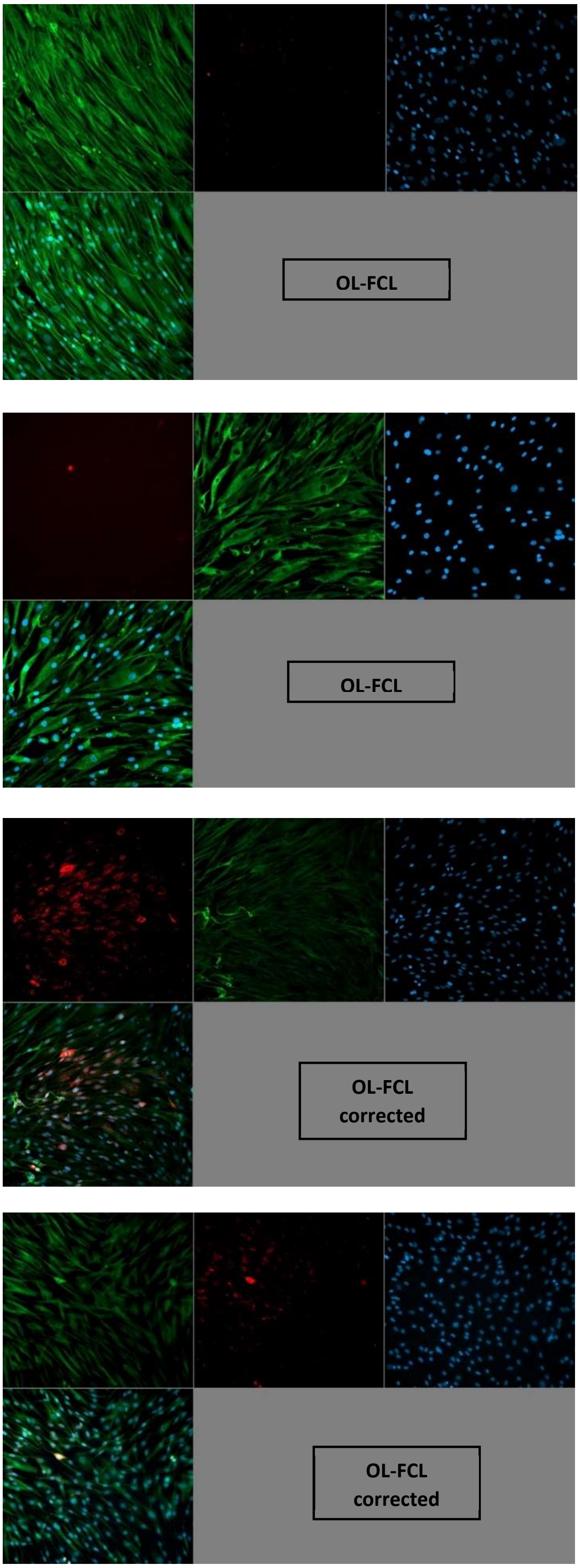

Figure 7:

FA-C fibroblasts (untreated)

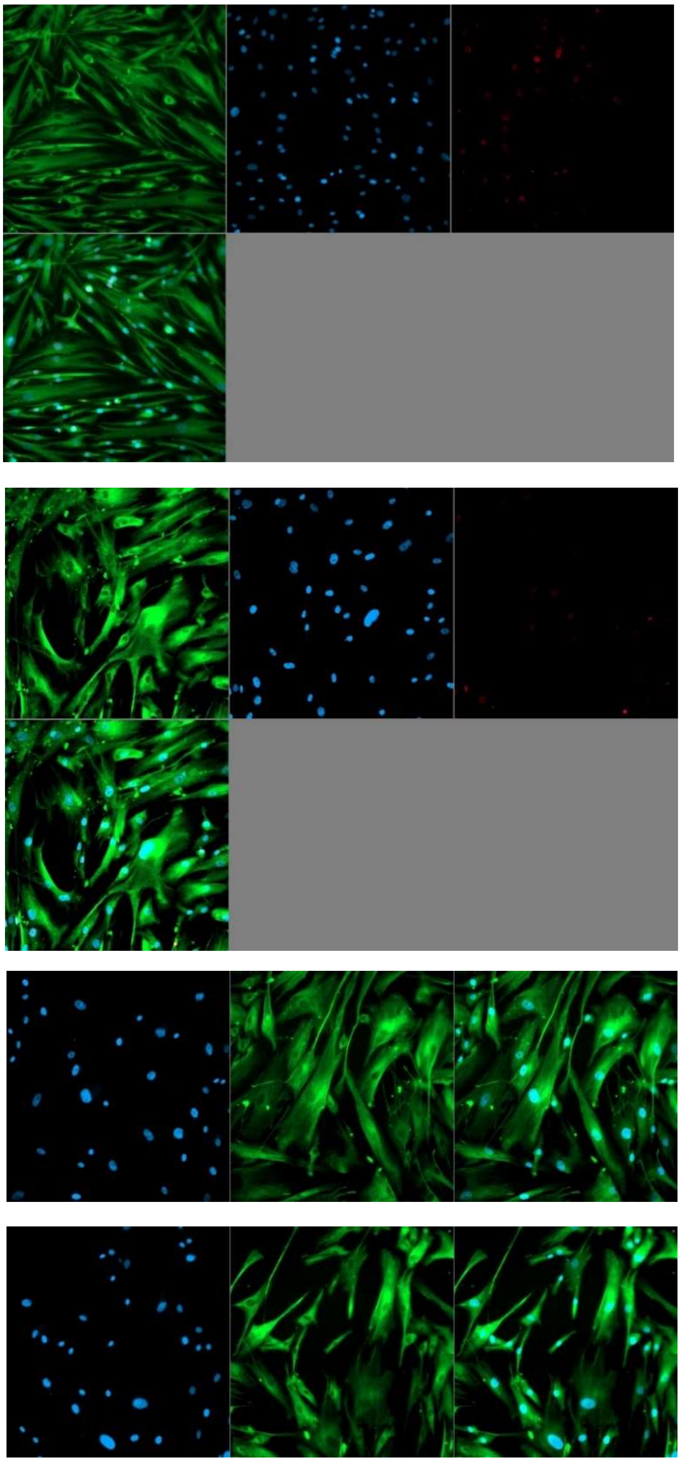

FA-C fibroblasts + ataluren 5μM (24h)

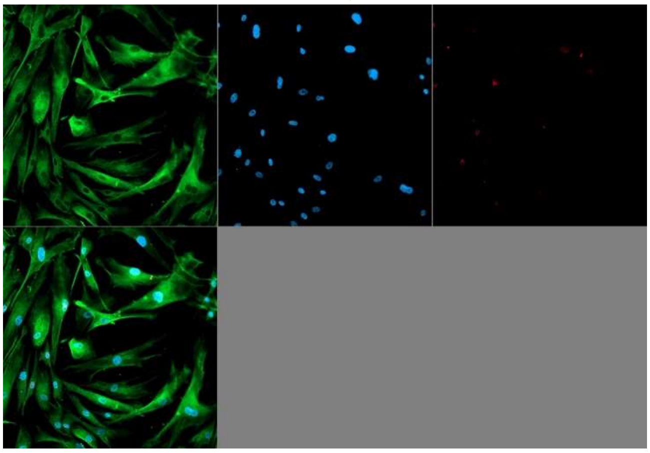

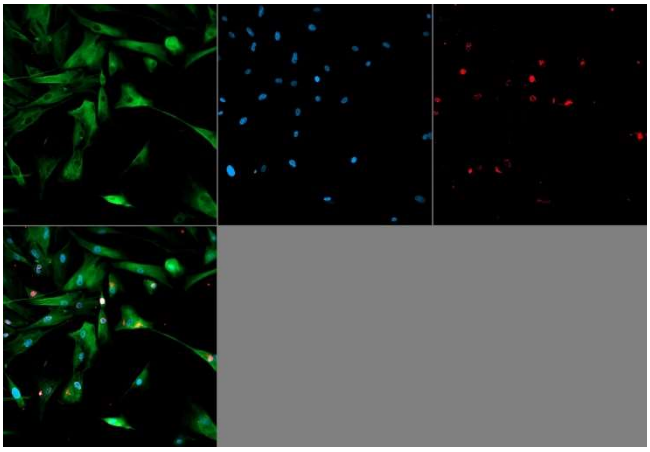

FA-C fibroblasts + amlexanox 25μM (24h)

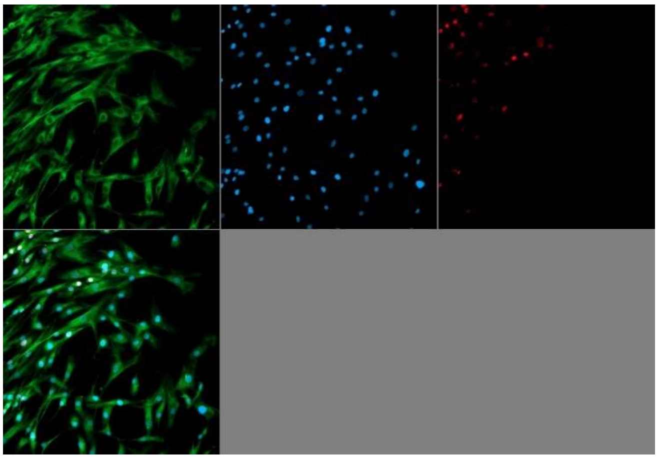

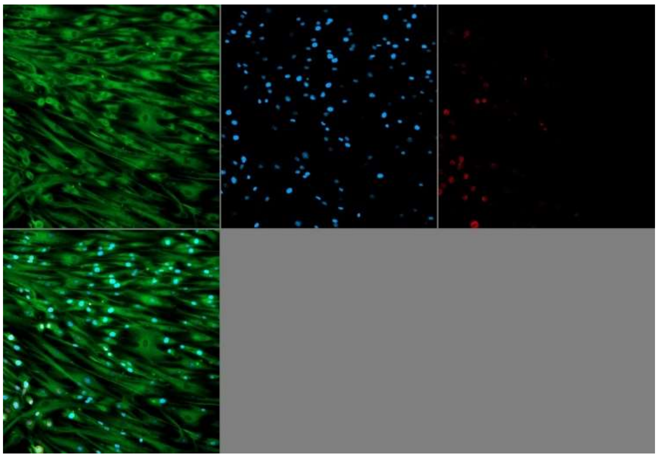

Supplementary Figure 1:

A	*FANCA*	31,38	31,49	31,28	32,15	31,75	30,35
		30,60	31,78	32,12	31,64	30,92	30,16
	sum	61,98	63,27	63,40	63,79	62,67	60,51
	mean	30,99	31,64	31,70	31,90	31,34	30,26
	st dev	0,55	0,21	0,59	0,36	0,59	0,13
	GAPDH	18,18	17,82	18,04	17,70	17,94	17,07
		18,03	18,02	17,82	17,75	18,06	17,01
	sum	36,21	35,84	35,86	35,45	36,00	34,08
	mean	18,11	17,92	17,93	17,73	18,00	17,04
	st dev	0,11	0,14	0,16	0,04	0,08	0,04
		**CTL NT**	**FA-A NT**	**FA-A + ATA**	**FA-A NT**	**FA-A + ATA**	**FA-A + ATA**
	DCt	12,89	13,72	13,77	14,17	13,34	13,22
		0,32	0,06	0,38	0,13	0,35	0,02
	st dev	0,56	0,25	0,61	0,36	0,59	0,14
	dct	12,89	13,72	13,77	14,17	13,34	13,22
		0,11	0,14	0,16	0,04	0,08	0,04
	**calibrator**	12,89	12,89	12,89	12,89	12,89	12,89
	st dev	0,56	0,56	0,56	0,56	0,56	0,56
	ddCT	0,00	0,82	0,88	1,28	0,45	0,33
		0,32	0,33	0,34	0,31	0,32	0,32
		0,57	0,58	0,58	0,56	0,57	0,56
		-0,57	0,25	0,30	0,72	-0,12	-0,24
		0,56	1,40	1,46	1,84	1,01	0,89
	max	1,49	0,84	0,81	0,61	1,09	1,18
	Expression	**1,00**	**0,56**	**0,54**	**0,41**	**0,73**	**0,80**
	min	0,68	0,38	0,36	0,28	0,50	0,54
		0,68	0,68	0,68	0,68	0,68	0,68
		1,47	1,47	1,47	1,47	1,47	1,47
			**-1,771535**	**-1,840375301**	**-2,42838977**	**-1,361314116**	**-1,252664439**

B	*FANCA*	32,93	33,47	33,32	28,49	30,81	31,18	30,03	31,13	31,19	28,12	31,20	31,59
		32,86	33,31	33,39	28,27	30,85	30,64	30,20	30,79	30,25	27,97	31,17	31,77
	sum	65,79	66,78	66,71	56,76	61,66	61,82	60,23	61,92	61,44	56,09	62,37	63,36
	mean	32,90	33,39	33,36	28,38	30,83	30,91	30,12	30,96	30,72	28,05	31,19	31,68
	st dev	0,05	0,11	0,05	0,16	0,03	0,38	0,12	0,24	0,66	0,11	0,02	0,13
	GAPDH	18,49	18,6	18,48	18,51	18,72	18,74	18,10	18,20	17,87	18,03	18,13	18,90
		18,5	18,92	18,24	18,43	18,64	18,43	18,20	18,12	17,88	17,87	18,17	18,50
	sum	36,99	37,52	36,72	36,94	37,36	37,17	36,30	36,32	35,75	35,90	36,30	37,40
	mean	18,50	18,76	18,36	18,47	18,68	18,59	18,15	18,16	17,88	17,95	18,15	18,70
	st dev	0,01	0,23	0,17	0,06	0,06	0,22	0,07	0,06	0,01	0,11	0,03	0,28
		**CTL NT**	**FA-A NT**	**FA-A+AMX**	**CTL NT**	**FA-A NT**	**FA-A+AMX**	**CTL NT**	**FA-A NT**	**FA-A+AMX**	**CTL NT**	**FA-A NT**	**FA-A+AMX**
	DCt	14,40	14,63	15,00	9,91	12,15	12,33	11,97	12,80	12,85	10,10	13,04	12,98
		0,00	0,06	0,03	0,03	0,00	0,19	0,02	0,06	0,44	0,02	0,00	0,10
	st dev	0,05	0,25	0,18	0,17	0,06	0,44	0,14	0,25	0,66	0,16	0,04	0,31
	dct	14,40	14,63	15,00	9,91	12,15	12,33	11,97	12,80	12,85	10,10	13,04	12,98
		0,05	0,25	0,18	0,06	0,06	0,22	0,07	0,06	0,01	0,11	0,03	0,28
	**calibrator**	14,40	14,40	14,40	9,91	9,91	9,91	11,97	11,97	11,97	10,1	10,1	10,1
	st dev	0,05	0,05	0,05	0,17	0,17	0,17	0,14	0,14	0,14	0,16	0,16	0,16
	ddCT	0	0,23	0,60	0,00	2,24	2,42	0,00	0,83	0,87	-0,01	2,94	2,88
		0,01	0,07	0,03	0,03	0,03	0,08	0,02	0,02	0,02	0,04	0,03	0,11
		0,07	0,26	0,18	0,18	0,18	0,28	0,16	0,15	0,14	0,20	0,16	0,32
		-0,07	-0,03	0,41	-0,18	2,06	2,14	-0,16	0,68	0,73	-0,20	2,77	2,56
		0,07	0,49	0,78	0,18	2,42	2,69	0,15	0,98	1,02	0,19	3,10	3,20
	max	1,05	1,02	0,75	1,13	0,24	0,23	1,12	0,62	0,60	1,15	0,15	0,17
	Expression	**1,00**	**0,85**	**0,66**	**1,00**	**0,21**	**0,19**	**1,00**	**0,56**	**0,55**	**1,00**	**0,13**	**0,14**
	min	0,95	0,71	0,58	0,88	0,19	0,15	0,90	0,51	0,49	0,88	0,12	0,11
		0,97	0,97	0,97	0,89	0,89	0,89	0,91	0,91	0,91	0,90	0,90	0,90
		1,04	1,04	1,04	1,13	1,13	1,13	1,10	1,10	1,10	1,12	1,12	1,12
			**-1,1728349**	**-1,510472586**		**-4,72**	**-5,33**		**-1,78**	**-1,83**		**-7,65**	**-7,36**

C	*FANCF*	26,73	26,82	26,8	26,73	26,65	26,85	29,38	29,74	29,9
		26,66	26,8	26,78	26,82	26,55	27,16	29,68	29,72	29,59
	sum	53,39	53,62	53,58	53,55	53,20	54,01	59,06	59,46	59,49
	mean	26,70	26,81	26,79	26,78	26,60	27,01	29,53	29,73	29,75
	st dev	0,05	0,01	0,01	0,06	0,07	0,22	0,21	0,01	0,22
	GAPDH	17,42	17,94	17,82	17,03	17,67	17,79	18,93	19,63	19,56
		17,36	17,94	17,85	17,1	17,63	17,86	19,04	19,36	19,51
	sum	34,78	35,88	35,67	34,13	35,30	35,65	37,97	38,99	39,07
	mean	17,39	17,94	17,84	17,07	17,65	17,83	18,99	19,50	19,54
	st dev	0,04	0,00	0,02	0,05	0,03	0,05	0,08	0,19	0,04
		**CTL NT**	**FA-F NT**	**FA-F+AMX**	**CTL NT**	**FA-F NT**	**FA-F+AMX**	**CTL NT**	**FA-F NT**	**FA-F+AMX**
	DCt	9,31	8,87	8,96	9,71	8,95	9,18	10,55	10,24	10,21
		0,00	0,00	0,00	0,01	0,01	0,05	0,05	0,04	0,05
	st dev	0,07	0,01	0,03	0,08	0,08	0,22	0,23	0,19	0,22
	dct	9,31	8,87	8,96	9,71	8,95	9,18	10,55	10,24	10,21
		0,07	0,01	0,03	0,08	0,08	0,22	0,23	0,19	0,22
	**calibrator**	9,31	9,31	9,31	9,71	9,71	9,71	10,55	10,55	10,55
	st dev	0,07	0,07	0,07	0,08	0,08	0,08	0,23	0,23	0,23
	ddCT	0	-0,43	-0,35	0,00	-0,76	-0,53	0	-0,31	-0,34
		0,01	0,00	0,00	0,01	0,01	0,06	0,10	0,09	0,10
		0,09	0,07	0,07	0,11	0,11	0,24	0,32	0,30	0,32
		-0,09	-0,50	-0,42	-0,11	-0,87	-0,77	-0,32	-0,61	-0,65
		0,09	-0,37	-0,28	0,11	-0,65	-0,29	0,32	-0,01	-0,02
	max	1,07	1,42	1,34	1,08	1,83	1,70	1,25	1,52	1,57
	Expression	**1,00**	**1,35**	**1,27**	**1,00**	**1,69**	**1,44**	**1,00**	**1,24**	**1,26**
	min	0,94	1,29	1,21	0,92	1,57	1,22	0,80	1,01	1,01
		0,96	0,96	0,96	0,95	0,95	0,95	0,86	0,86	0,86
		1,05	1,05	1,05	1,06	1,06	1,06	1,17	1,17	1,17

Supplementary Figure 2:

S2.A

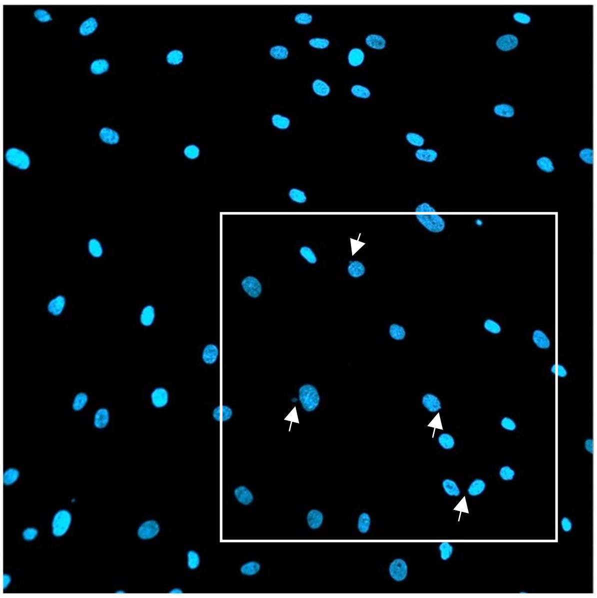

S2.B

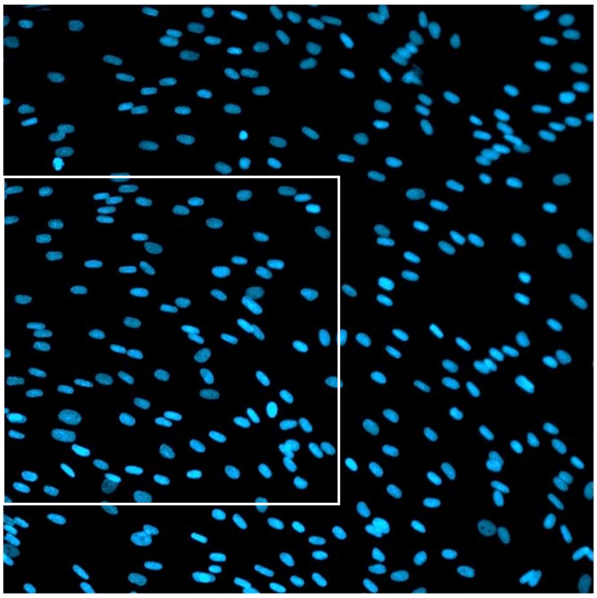

Supplementary Figure 3:

S3.A

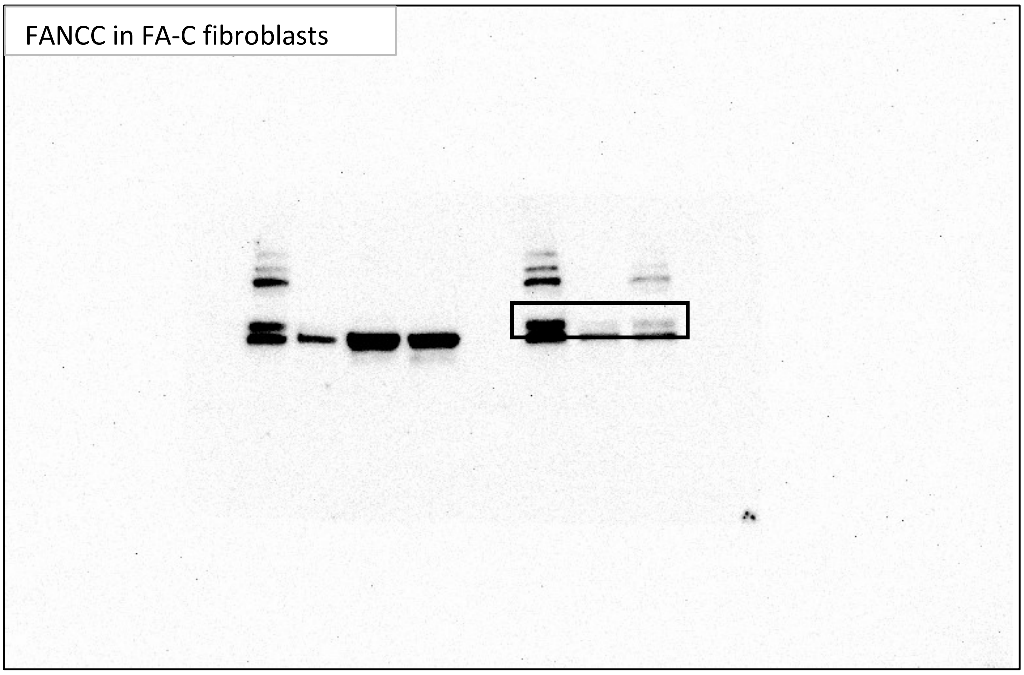

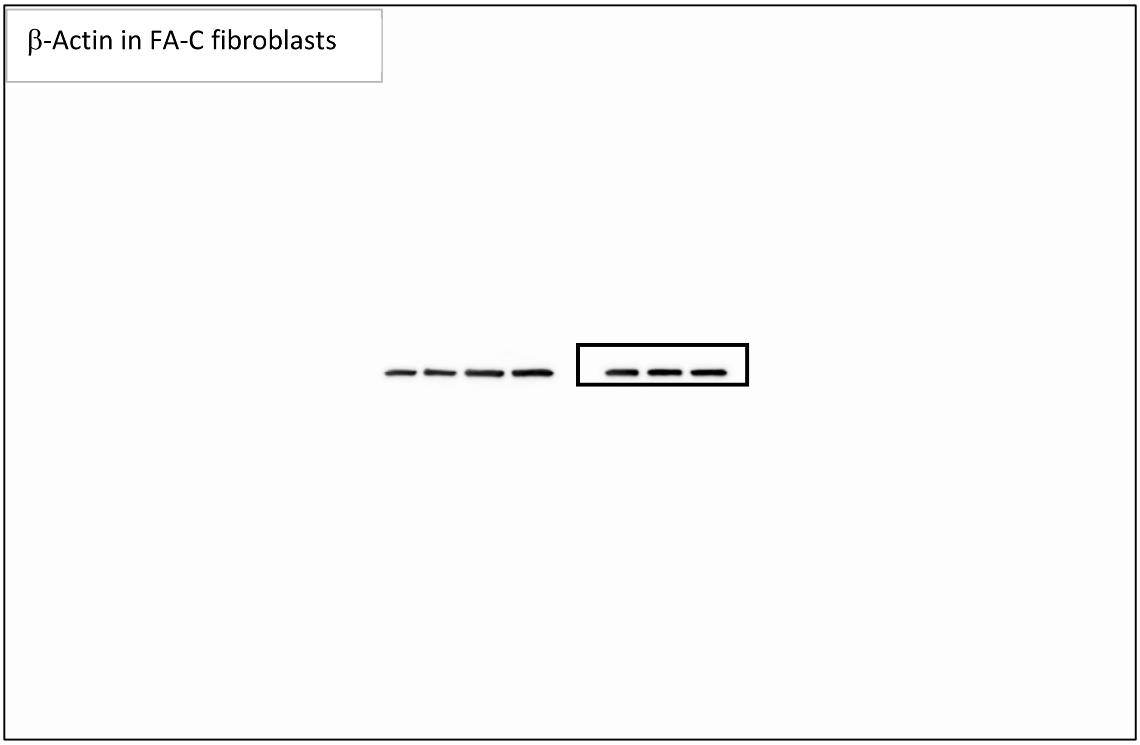

S3.B

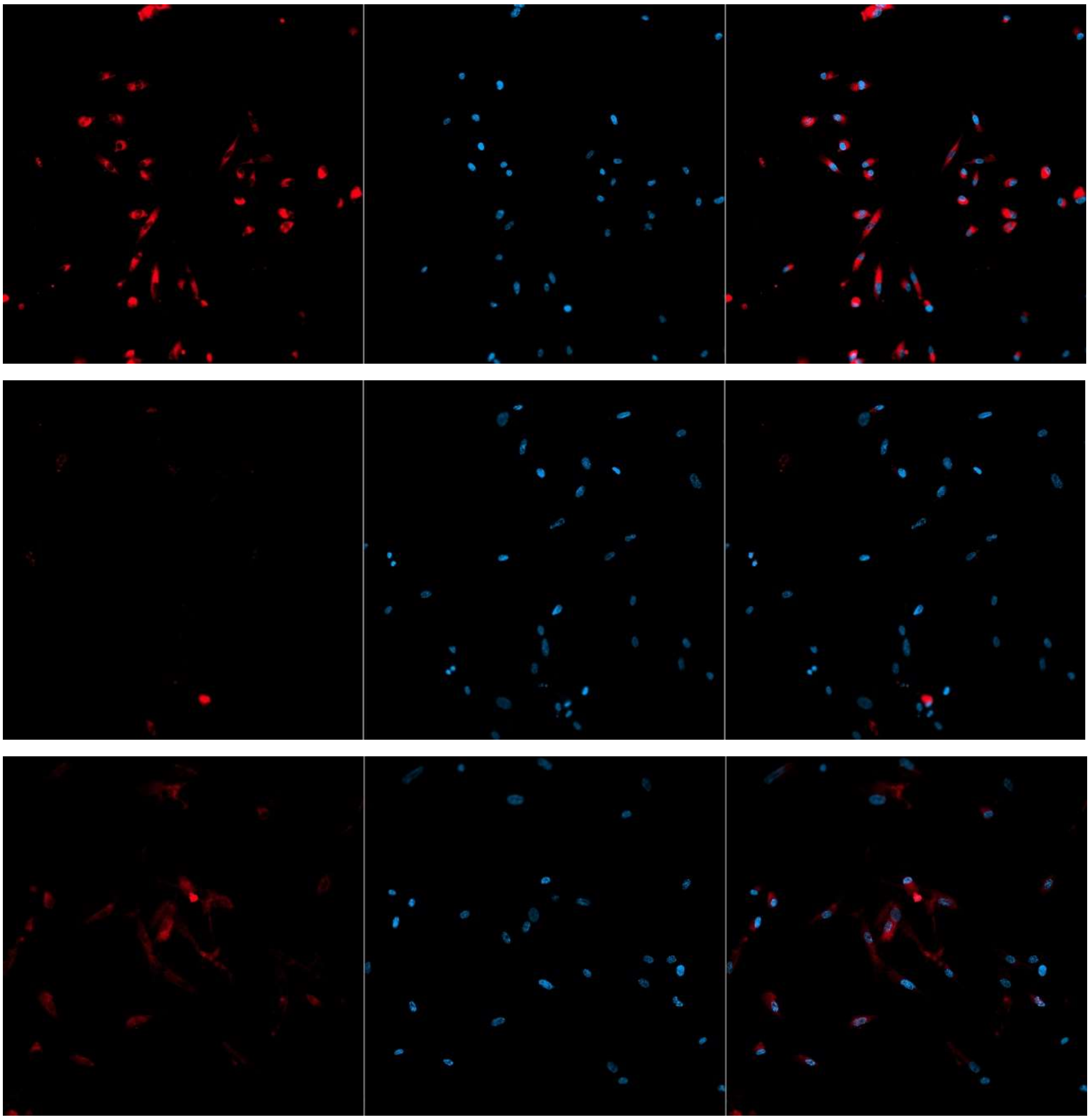

Supplementary Figure 4:

S4.A

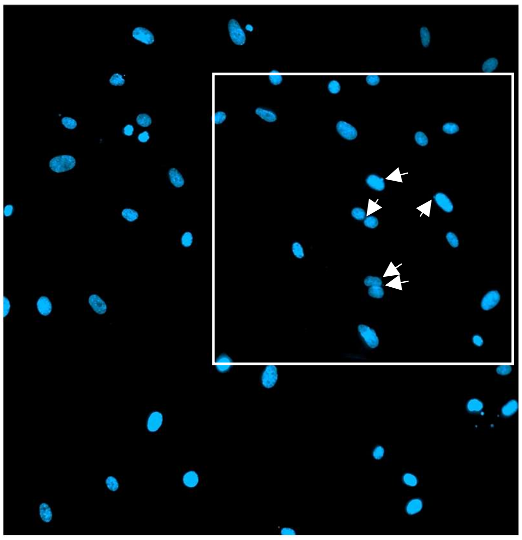

S4.B

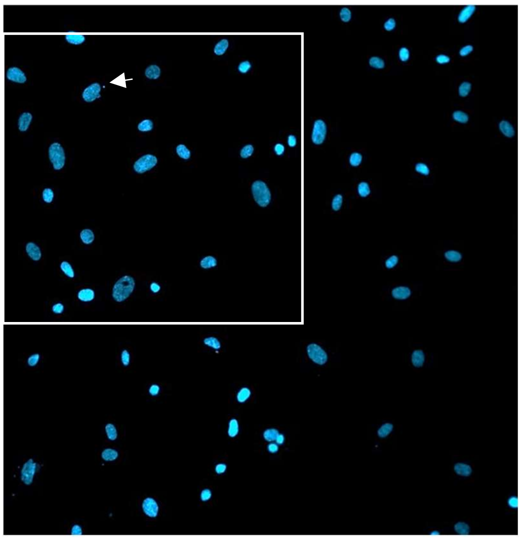

S4.C

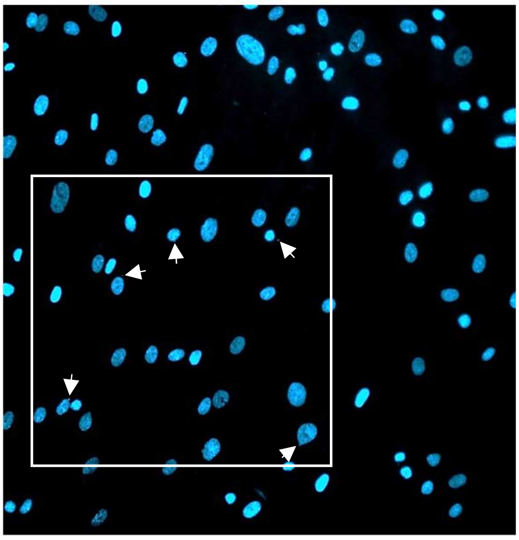

<u>Immunofluorescence negative controls</u> (FITC, APC, DAPI) to main figures 6 and 7 and supplementary figure 3

OL-FCL

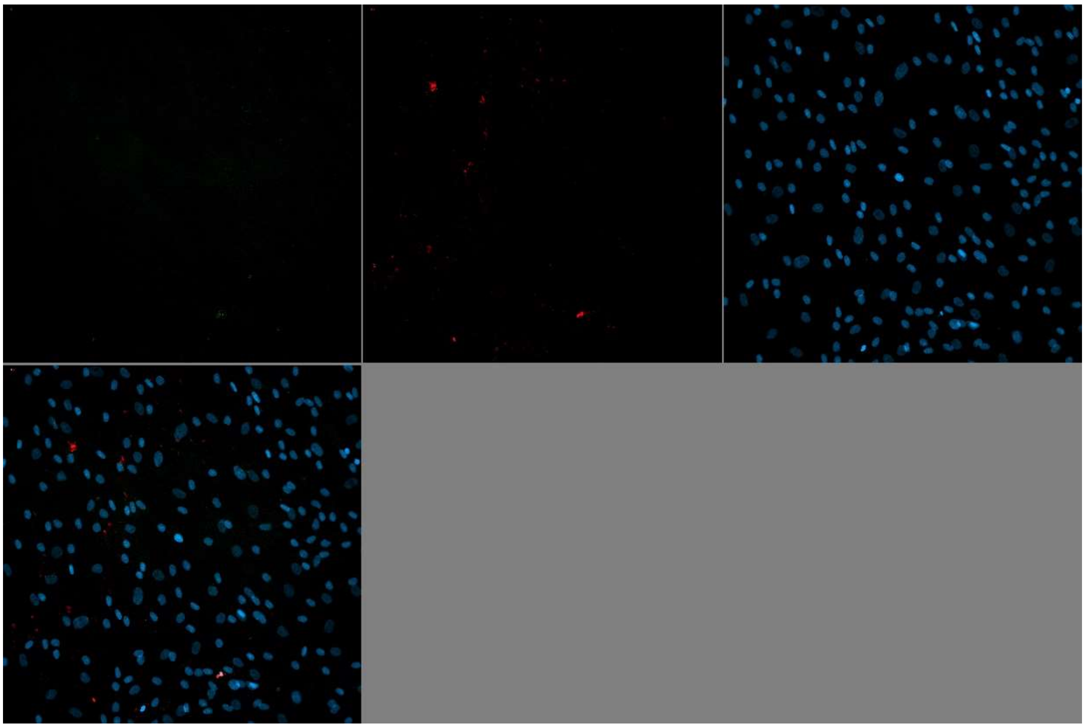

OL-FCL-corr

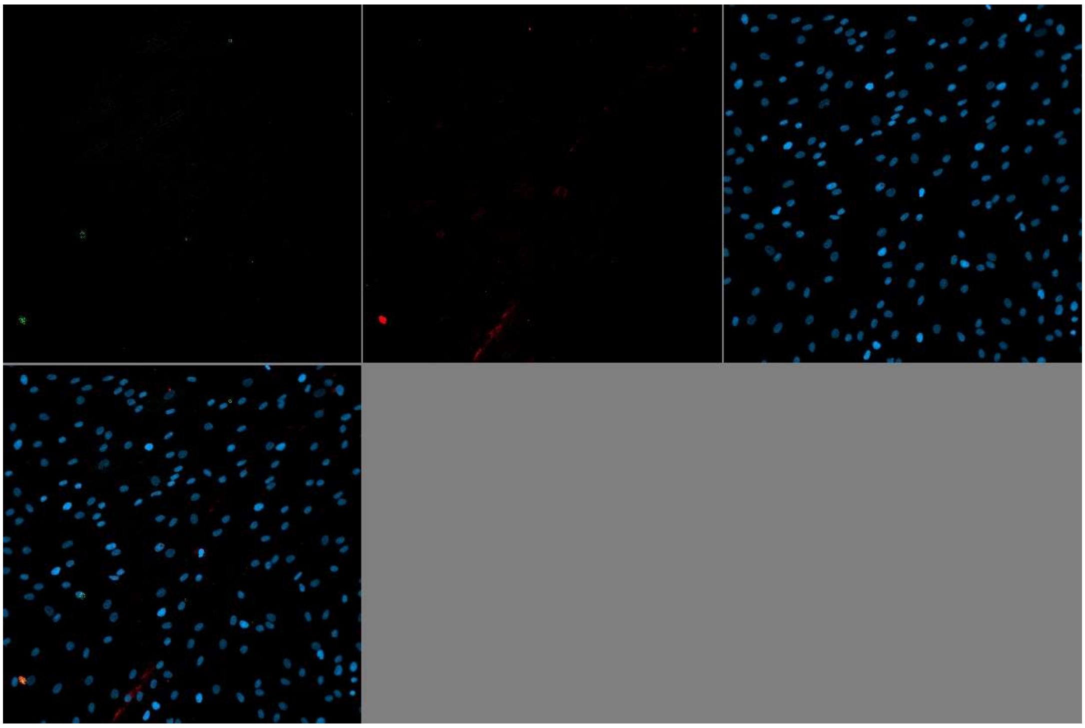

FA-C

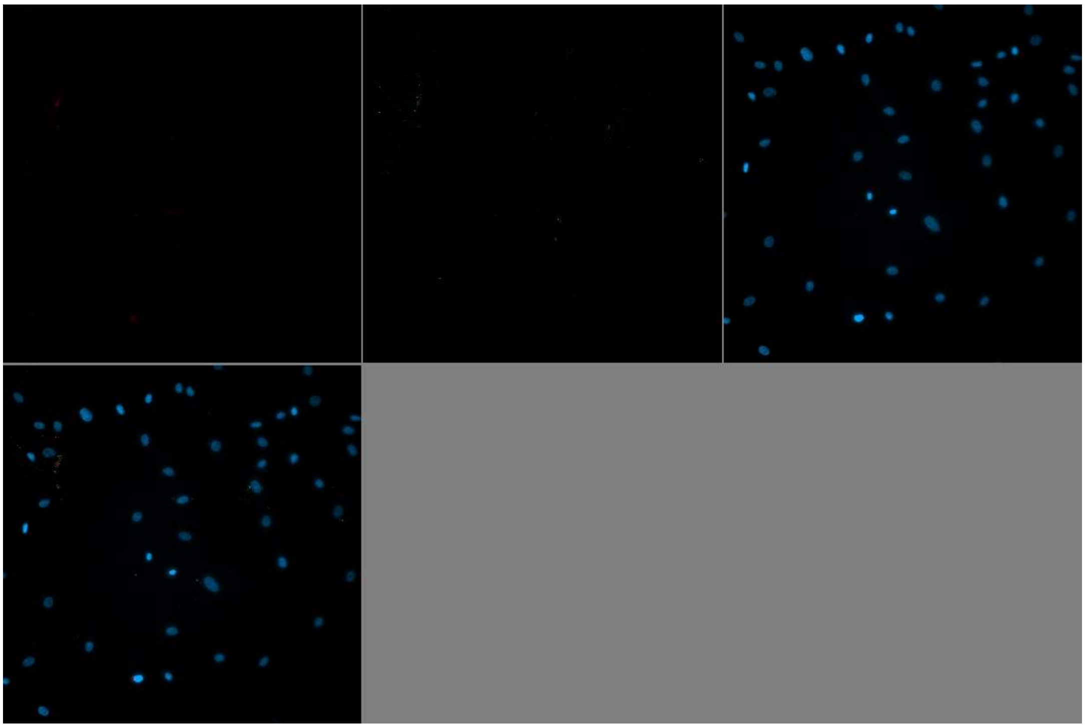

CTL

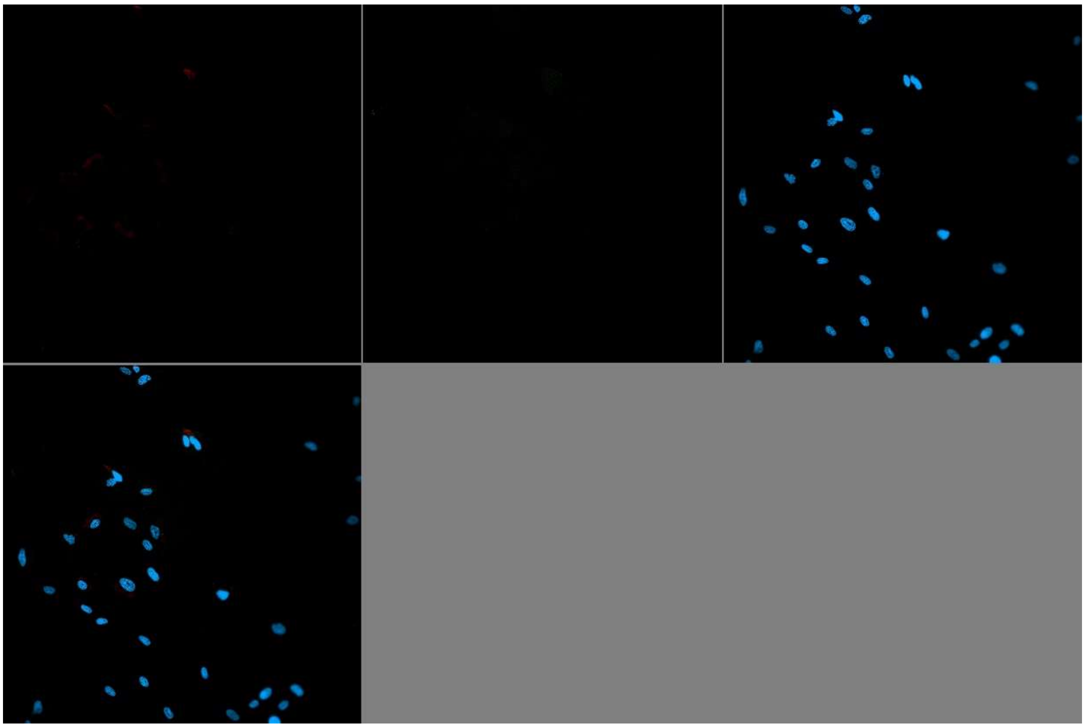

Supplement: Supplementary file 2 — Original Data [file 41420_2025_2571_MOESM2_ESM.pdf]
